# Benthic fauna declined on a whitening Antarctic continental shelf

**DOI:** 10.1038/s41467-020-16093-z

**Published:** 2020-05-06

**Authors:** Santiago E. A. Pineda-Metz, Dieter Gerdes, Claudio Richter

**Affiliations:** 1 Alfred-Wegener-Institut Helmholtz-Zentrum für Polar- und Meeresforschung, 27568 Bremerhaven, Germany; 2 Universität Bremen (Fachbereich 2, Biologie/Chemie), 28334 Bremen, Germany

**Keywords:** Community ecology, Zoology

## Abstract

Ice retreat in West Antarctica and Antarctic Peninsula has led to important changes in seafloor communities and gains in benthic blue carbon. In most of the Antarctic, however, sea ice increased between the 1970s and 2014, but its effects on the benthos remain largely unexplored. Here, we provide a 1988–2014 record of macro- and megafauna from the north-eastern Weddell Sea shelf, where benthic biomass decreased by two thirds and composition shifted from suspension feeders to deposit feeders. Concomitant increases in sea-ice cover suggest a reduced flux of primary production to the benthos. As benthic communities are major repositories for Antarctic biodiversity and play an important role in biogeochemical cycling, the observed changes have far-reaching consequences for the Antarctic ecosystem and its feedback to the climate system. The findings underscore the importance of long-term ecological monitoring in a region vulnerable to warming and ice-shelf collapse.

## Abstract (web summary)

Sea-ice cover in Antarctica has increased over the last decades and reached a maximum in 2014. Here, the authors report strong declines in zoobenthic biomass and abundance and changes in community composition on the NE Weddell Sea shelf over 26 years, with implications for blue carbon and biochemistry in a globally important marine region.

## Introduction

Antarctica harbours a unique benthic fauna displaying unusually high levels of biodiversity, endemism and biomass^1–5^. Antarctic benthos evolved in cold isolation, leading to life-forms characterized by low temperature adaptation, stenothermia, long generation time, late maturity age and brooding as prevalent reproductive strategy^1,6–9^. Extreme seasonal and interannual variations in food availability have favoured the capacity to maintain metabolic activity during periods of food scarcity^10,11^. Icebergs scouring the seafloor are a notorious source of disturbance creating large variations in abundance, biomass and species numbers on small spatial scales^12,13^.

The extreme patchiness, slow growth of most organisms^1,14^, spatio-temporal heterogeneities in sampling effort, inconsistencies in sampling equipment and methods and scarcity of repeated measurements make it difficult to detect temporal changes in Antarctic benthic communities. As a result, only few studies conducted around the Southern Ocean have shown climate-related changes in benthic communities and key benthic groups. Most studies on the Antarctic benthos in a changing climate are in response to glacier, sea ice, anchor ice retreat, increased iceberg scouring and ice-shelf collapse^15–19^, establishing a link between pelagic productivity and carbon stored by benthos (blue carbon)^18,20,21^. As opposed to the Arctic, where sea-ice has been plummeting over the last decades^22^, two thirds of the Antarctic including the Weddell Sea, the East Antarctic shelf and the Ross Sea have seen increasing trends of sea-ice cover over the satellite instrumental period from 1979 up to a maximum in 2014^23,24^, but the response of the benthos to this larger whitening region of the Antarctic is so far unexplored. Long-term studies from the shallow part of McMurdo Sound in the Ross Sea, showed decadal changes in the benthic community^25^ and in the growth and recruitment of sponges^26,27^, likely in response to changes in local productivity due to grounded icebergs. The only available data for the eastern Weddell Sea continental shelf shows a positive relationship between bryozoan growth and open water extent (and hence productivity) in an area of increasing sea-ice cover^18^. Given the absence of systematic repeated samplings with standardized methods, quantitative community scale time series data on the benthos from the Antarctica’s whitening deep continental shelves are so far lacking.

Here, we present a 26-year record of macrobenthic biomass and abundance off Kapp Norvegia/Auståsen (KNA) in the north-eastern Weddell Sea, and its associated changes with increasing summer sea-ice cover during the last decades^28–30^. KNA is considered a gate of entry for icebergs into the Weddell Sea^31^ and a hotspot of iceberg grounding^20^. As sea-ice cover is inversely related to productivity^32^ and organic material reaching the seafloor^33^, while icebergs act antagonistically reducing benthos by scouring^12,16,34^ and consolidating sea-ice between grounded icebergs^25^, our data set provides an opportunity to assess benthic biological responses to a whitening Antarctica.

**Fig. 1:**
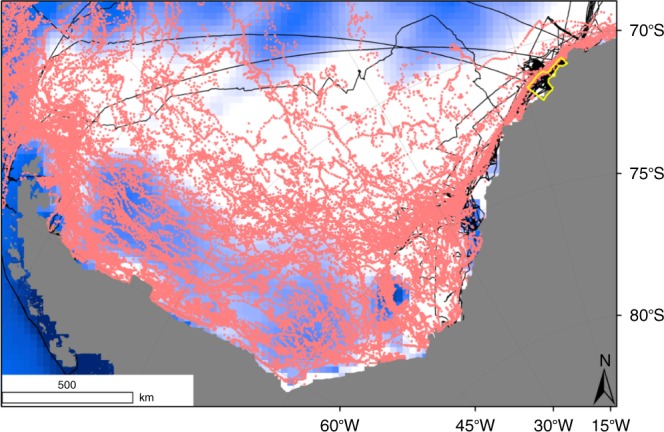
Map of the Weddell Sea. The map includes position of giant icebergs (pink dots), tracks of R/V *Polarstern* cruises to the Weddell Sea providing material for this study (black lines), and areas were sea-ice cover has increased (white) and decreased (blue). The yellow polygon, drawn as a polygon shapefile using the ArcGIS 10.x software, represents the study area off Kapp Norvegia/Auståsen.

KNA represents the intersection of eight ship-based expeditions between 1988 and 2014 (Fig. 1). Here, 59 multibox corer deployments were carried out, processed and analysed by the same protocol and staff. Additionally, we analyse 98 imagery transects (see Methods), to assess the megafauna, which is not quantitatively represented in the box cores, and whose complexity varies in response to iceberg disturbance. To represent productivity and iceberg influence, we consider data on sea-ice cover and iceberg area available from several data bases (see Methods). We relate these data to benthic variations and the main southern hemisphere climatic pattern, the Southern Annular Mode (SAM)^28,30,35,36^. Here we show temporal changes in benthic macrofauna abundance and biomass, megabenthic 3D complexity, and benthic community composition. Additionally, we relate the faunal changes to concomitant changes in sea-ice related productivity, and variation of iceberg area during our study period.

## Results

### Loss of benthic abundance and biomass (blue carbon)

**Fig. 2:**
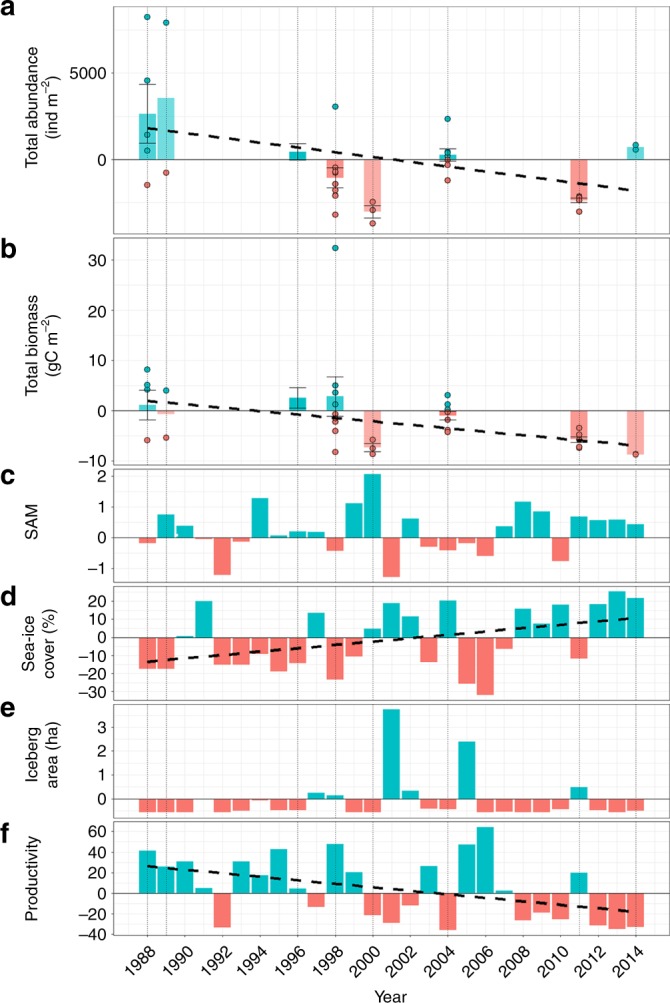
Temporal changes in macrobenthic and environmental characteristcs between 1988–2014. Changes are given for macrobenthos abundance (**a**), macrobenthos biomass (**b**), summer Southern Annular Mode index (SAM, **c**), sea-ice cover (**d**), iceberg area (**e**) and productivity (**f**) for the time period 1988–2014. Values are given as deviations from the long-term average. For SAM, sea-ice cover, iceberg area and productivity *n* = 1 for all years. Dashed lines represent significant Pearson linear regressions (*p* < 0.05, permutations = 9999). Dots represent independent multibox corer deployments for 1988 (*n* = 5), 1989 (*n* = 2), 1996 (*n* = 24), 1998 (*n* = 9), 2000 (*n* = 3), 2004 (*n* = 8), 2011 (*n* = 6) and 2014 (*n* = 2); note that only *n* ≤ 10 are displayed. Bars represent mean values, black whiskers represent standard errors. For abundance and biomass the shading denotes *n* (stronger colours represent larger *n*). Underlying data are provided as a Source data file.

Macrobenthic abundance and biomass dropped by half and two thirds of the late 80s values, respectively, in the second half of the time series (Fig. 2a, b; Table 1). The southern annular mode (SAM) switched from a more negative to a slightly more positive phase (Fig. 2c) during this period, along with large increases in sea-ice cover (Fig. 2d), no trend in iceberg area (Fig. 2e), and a pronounced drop in productivity (Fig. 2f) were observed. The largest abundance and biomass changes, occurred at the turn of the millennium, coinciding with the overall SAM maximum. Abundance and biomass of feeding guilds followed the pattern for the total community (Supplementary Figs. 1 and 2).

**Table 1:** Benthic mean abundance and biomass for Kapp Norvegia/Auståsen.

Year	Number of stations	Mean Abundance (ind m^−2^)	Mean Biomass (gC m^−2^)
1988	5	6388 ± 1710	9.90 ± 2.95
1989	2	7304 ± 4354	8.04 ± 4.72
1996	24	4177 ± 465	11.29 ± 2.04
1998	9	2674 ± 587	11.57 ± 3.93
2000	3	697 ± 359	1.40 ± 0.83
2004	8	3989 ± 354	7.79 ± 0.88
2011	6	1364 ± 135	2.98 ± 0.61
2014	2	4431 ± 130	0.02 ± 0.02

Mean values derived from all independent multibox corer stations of each campaign, plus/minus represents the standard error.

**Fig. 3:**
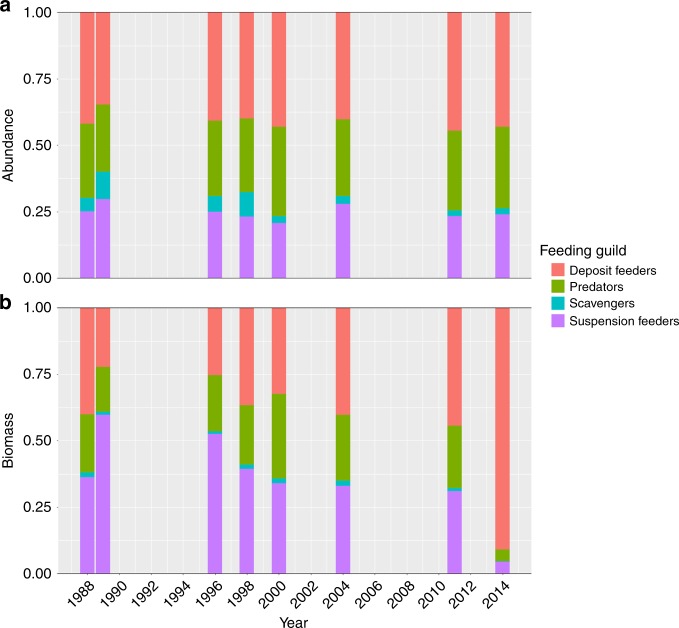
Temporal changes of macrobenthic composition between 1988–2014. Temporal changes are given for the contribution of each feeding guild to macrobenthic abundance (**a**) and biomass (**b**). Each coloured bar represents the average abundance and biomass proportion of each feeding guild. Averages were calculated based on data from independent multibox corer deployments for 1988 (*n* = 5), 1989 (*n* = 2), 1996 (*n* = 24), 1998 (*n* = 9), 2000 (*n* = 3), 2004 (*n* = 8), 2011 (*n* = 6) and 2014 (*n* = 2). Underlying data are provided as a Source data file.

While total macrofauna abundance decreased during our study period (Fig. 2a), the relative contribution of feeding guilds to total macrofauna abundance remained fairly constant (Fig. 3a). This suggests similar responses by different functional groups of the macrofauna inhabiting KNA and, thus, a high interconnectivity between individuals of the community. Contrastingly, the decrease in total macrofauna biomass (Fig. 2b) was accompanied by a shift from a suspension feeder dominated community in the 80s and 90s to one dominated by deposit feeders in the new millennium (Fig. 3b). As benthic suspension feeders depend directly on the vertical flux of organic matter, their decline provides additional evidence for a reduced productivity in response to increased sea-ice cover.

**Fig. 4:**
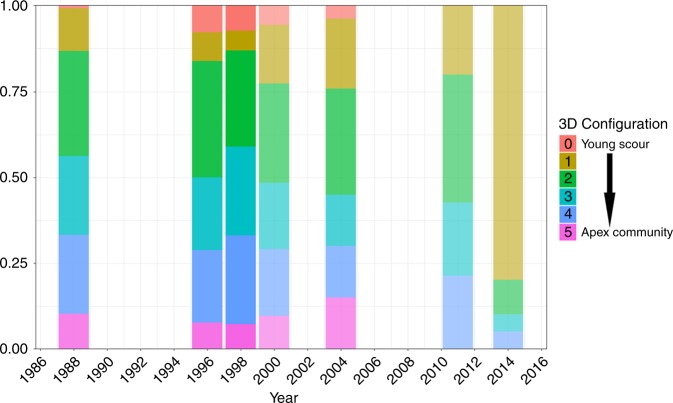
Temporal changes in the frequency of megafaunal stage of recolonization following putative iceberg disturbance. Categories ranging from flat (no megafauna) to fully three-dimensional apex communities (see Methods) were applied to independent imagery transects obtained during 1988 (*n* = 12), 1996 (*n* = 27), 1998 (*n* = 33), 2000 (*n* = 9), 2004 (*n* = 11), 2011 (*n* = 3) and 2014 (*n* = 3). Shading denotes *n* (stronger colours represent larger *n*; see Supplementary Table 3). Underlying data are provided as a Source data file.

The megafauna showed a similar decline over the study period, with a decreasing proportion of fully mature apex communities (see Methods) and an increase in the proportion of young successional community stages (Fig. 4). Large Antarctic glass sponges are the main component of benthic communities in the Weddell Sea^1–3,5^. These organisms enhance biodiversity and provide refuge for other organisms which, in turn, increases the local benthic biomass. Thus, the decrease of total macrobenthic biomass (Fig. 2b) in addition to the loss of 3D complexity can be associated to biomass losses of large foundation species (e.g. sponges), which are the largest suspension feeders.

### Environmental factors and benthos: trends and relations

A positive phase of the SAM is associated with higher summer sea-ice concentrations in the eastern Weddell Sea^28^, a lower areal extent and duration of productive polynya areas and a lower vernal supply of pelagic food to the benthos^32^. Model-based studies suggest a delayed response of sea-ice cover to SAM variation^37,38^. Based on regression analyses, we were able to confirm SAM variations to significantly affect sea-ice cover changes with a delay of one year (Pearson *r*^2^ = 0.404; *p*-value = 0.0165; Permutations = 9999). The negative correspondence between SAM and productivity suggests that ice-free days and ice-free area dominate over other factors governing pelagic productivity in Antarctica (e.g. nutrients, photo-synthetically available radiation, sea surface temperature, continental shelf width, basal melt rate)^32^.

**Fig. 5:**
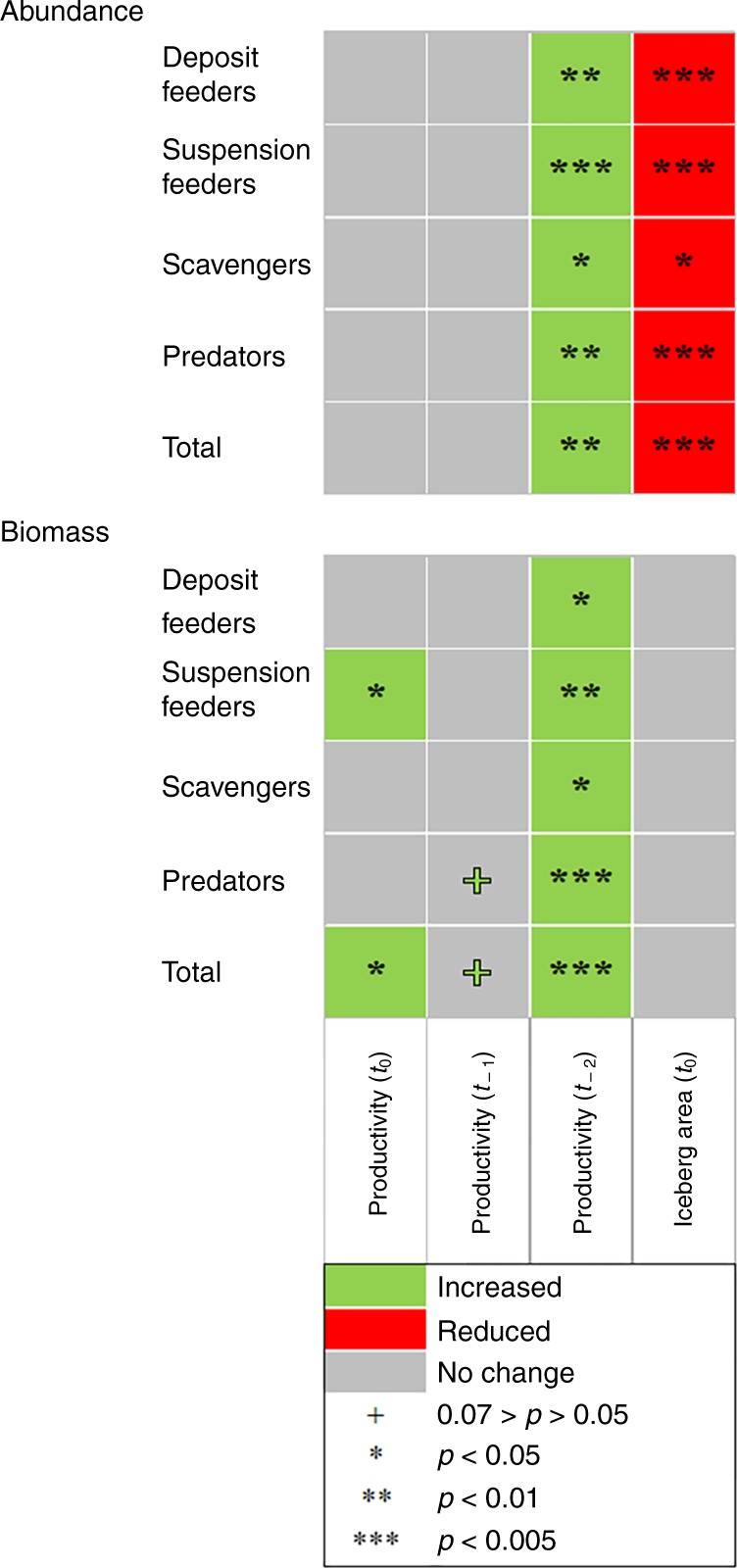
Influence of productivity and iceberg area on total benthic abundance and biomass, and abundance and biomass of major feeding guilds. Spearman correlations were calculated considering all independent multibox corer deployments (*n* = 59), and 9999 permutations. Significant correlations (*p* < 0.05) are represented by coloured boxes. *p*-values shown with + represent a statistical trend^69^, the colours represent positive (green) and negative (red) correlation statistic values, respectively. Underlying data are provided as a Source data file.

Correlation analyses showed positive relationships between benthic abundance and biomass and pelagic productivity, both for the total community as well as for the major feeding guilds, (*p* < 0.05, permutations = 9999; green areas in Fig. 5). Biomass of suspension feeders showed an immediate response to increases in productivity (year *t*_0_; *p* < 0.05, permutations = 9999), commensurate with their direct dependence on fresh organic matter. The instantaneous response of the benthos supports earlier findings of rapid sponge growth^17,26^ overturning the long-held paradigm of slow growth in polar waters^9^. The observed coupling of macrobenthic biomass and productivity also supports the hypothesis that Antarctic benthos is food limited^39^. Deposit feeders and higher trophic levels (scavengers and predators), by contrast, showed a delayed biomass response (years *t*_1_ and *t*_2_, Fig. 5). The lack of immediate response suggests a decoupling from primary production at the temporal scales investigated. A plausible explanation is that the energy for these feeding guilds becomes available only after it settles on the seabed (deposit feeders) or passes through other organisms (scavengers and predators)^11,33^. Abundances showed a 2-years phase-shift for all groups (Fig. 5), which would be consistent with the rather long time it takes for gonad development, reproduction and recruitment^40,41^.

We found inverse correlations between total benthic abundance and iceberg area, as well as abundance of all feeding guilds (*p* < 0.05, permutations = 9999; Fig. 5). Biomass appeared to be independent of iceberg area (Fig. 5). As opposed to shallow coastal areas, where increased frequency and mobility of icebergs resulted in increased scouring on the shelf^16,42^, the changes in icebergs in our study were not associated with inverse changes in either biomass or megabenthic 3D complexity.

## Discussion

Our study, spanning more than a quarter of a century, is the first, to the best of our knowledge, showing quantitative benthic community changes over the full depth of the continental shelf. It is also, to our knowledge, the first to examine benthic changes in a region of the Antarctic that has been whitening until very recently, thus providing an important test of the proposed links between sea-ice cover and iceberg area and benthic abundance and biomass^43^. Because most of the Antarctic continental shelf has seen a positive trend in sea-ice extent over 40 years of satellite observations until 2014^28,44^, an overall decrease in blue carbon can be expected. If corroborated by geochemical evidence, the extrapolation of our findings to the total Antarctic continental shelf would imply an overall decrease in Antarctic zoobenthic blue carbon storage of 31.3 × 10^6^ t km^−2^ during that period, contrasting the situation described for the West Antarctic and Antarctic Peninsula regions where blue carbon has been increasing in response to climate change^17,18,20,21^.

The end of our study marks the possible end of three decades of increasing Antarctic sea-ice extent with a record high in 2014 followed by a precipitous decline to a record low only three years later^23^. It is not clear to date, how the trends will develop. There are indications for a turning point^24^, and a model for the eastern Weddell Sea predicts a decrease of sea-ice cover in the coming decades^44^. A decrease in sea-ice would lead to an increase in productivity, flux of organic matter to the benthos and, ultimately, in benthic abundance and biomass. A change from a whitening to a blueing Weddell and Ross Seas would roughly double the carbon sink initiated in the Bellingshausen/Amundsen continental shelf area, providing an important negative feedback to climate change^18,20,21^. However, carbon supply is only one of many factors limiting life on the Antarctic seafloor. Most of the few Antarctic benthic species to be investigated are stenothermal with narrow thermal windows^7,8,38^ showing only very limited potential to cope with the 1–2 °C temperature rises^7,8,41,45^, which at least for the Weddell Sea, already occur in the form of warm water intrusions^46^. Further temperature increases are predicted for the Weddell Sea in the coming decades^47^. The biological responses to the physico-chemical environment under different climate scenarios are complex^48^ and require an integrated and sustained effort to continue observations over time in this part of the planet that is critically vulnerable to climate change^49^.

## Methods

### Study area, benthic abundance, biomass and 3d configuration

Research was carried out in the Kapp Norvegia/Auståsen (KNA) region (Fig. 1), as delimited by previous studies conducted there^50,51^. To calculate the approximate area of KNA we constructed a polygon in the GIS environment with an approximate area of 9180 km^2^.

Benthic total abundance and biomass, and abundance and biomass data of 35 benthic taxa correspond to stations of the R/V *Polarstern*^52^ cruises ANT-VI/3. VII/4, XIII/3, XV/3, XVII/3, XXI/2, XXVII and PS82 (see https://www.pangaea.de/expeditions/cr.php/Polarstern for cruise reports), which represent the years 1988, 1989, 1996, 1998, 2000, 2004, 2011 and 2014, respectively. Only shelf stations were selected, i.e. all those stations located within 100–700 m depth, based on the definition of Antarctic shelves^1,33^. We excluded stations that were artificially disturbed in the long-term “BEnthic Disturbance EXperiment” (BENDEX)^53^, leaving a total of 59 multibox corer^54^ stations with a total 307 cores for the present study (Supplementary Table 1; note that for ANT-XXI/2 only the pooled values for the stations were available). The multibox corer was equipped with 9 cores, from which 1 to 8 were analysed for each station. Each core was 20 × 12 × 45 cm (L × W × D), providing an approximate total of 150 kg of sediment sample per station^54^. Sample size was adequate for the epi- and endobenthic macrofauna, but too small for the large megafauna (e.g. hexactinellid sponges)^55^, which was excluded from the analyses and categorized separately (see below). Sediments were sieved through 0.5 mm mesh and the organisms retained on the sieve were fixed in 5% borax-buffered formaline and stored until microscopic enumeration and biomass analysis, generally within a few months after collection^2^. The taxonomic units as defined by Pineda-Metz et al.^43^ were used to calculate abundance and biomass of four major feeding guilds: deposit feeders, suspension feeders, scavengers and predators. Separation of feeding guilds was done following the classification scheme shown in Supplementary Table 2. For colonial species (e.g. bryozoans and hydrozoans) abundance data were available only as presence/absence data.

To assess changes in the structural complexity of a benthic community from a flat (early successional stage) to a three-dimensional mature apex community dominated by megafauna (glass sponges, gorgonians), we analysed video- and photo-transects (i.e. imagery transects) from all expeditions, with the exception of 1989 for which no image data are available. Transects representing the years 1988, 1996, 1998, 2000, 2004 and one transect for 2014 (Supplementary Table 3), were extracted from the PANGAEA data repository.

Megafauna was described on a categorical basis using an ordinal scale ranging from 0 to 5. Category 0 represented a putative scour mark in its earliest stage of recolonization, characterized by a lack of megafauna and an abrupt change in benthic cover and size along the transect (equivalent to stage R0 in Gutt & Starmans^34^). Categories 1–5 represent later stages of colonization by Antarctic shelf megafaunal assemblages sensu Gili et al.^56^, of increasing three-dimensionality (see Fig. 1, in Gili et al.^56^). The frequency of all 3D configurations was assessed for all imagery transects, and was represented by five categories (from 0 to 4) to facilitate analysis. Each category represents the following proportions: 0 = absent/rare, 1 = uncommon, 2 = common, 3 = mainly, 4 = dominant. This first analysis gave a transect × 3D category table which was used with the prep.fuzzy function of the ade4 package for R^57^ to transform categorical values to percentage. These calculated percentages were used to estimate the average proportion of each 3D configuration category at each year.

### Environmental data and treatment

Environmental data were obtained from the National Sea Ice Data Center data repository^58^ (sea-ice cover), the OceanColor web data repository^59–62^ (Chl *a* and particulate organic carbon), Solar Geometry Calculator of the National Oceanic and Atmospheric Administration^63^ (NOAA), ALTIBERG Iceberg data base^64,65^ (average area of small icebergs), and the “The Climate Data Guide: Marshall Southern Annular Mode (SAM) Index (Station-based)^66^.

Monthly data of the Southern Annular Mode (SAM) index were obtained^66^ for all summers of the years 1986–2014. We considered as summer the period November to March, which represents the months when the local polynya is open^67^. Based on this, we calculated the summer SAM index as the average value considering the period November to March.

Daily sea-ice cover from the Sea ice Index provided by the National Snow and Ice Data Center was extracted for the summer months of the period 1986–2014^58^. The daily sea-ice cover data were used to calculate ice-free days in summer for each cell within the KNA polygon. Additionally, data from the Solar Geometry Calculator of the NOAA^63^ were used to calculate the proportion of a summer day with sun light during summers of the period 1986–2014.

Sea-ice and solar data were used to calculate a summer productivity index, referred in the main text as productivity. Arrigo et al.^32^ found polynya productivity to be directly related to mean daily photo-synthetically usable radiation, number of ice-free days, ice-free area, sea surface temperature, continental shelf width, and basal melt rate. Based on this, we calculated the productivity for the 1979–2014 period as the product of ice-free days and proportion of a summer day with sun light for all cells located in the KNA polygon (for a detailed description see Supplementary Method Productivity Raster Calculation).

To test if the calculated productivity works as a proxy for Chl *a* and particulate organic carbon concentration, we correlated productivity with summer Chl *a* and particulate organic carbon data obtained from the NASA Ocean Color web^59–62^ for our study area, and Chl *a* data for the whole Weddell Sea^67^. As all correlations were significant (*p* < 0.005; Supplementary Fig. 3) and had coefficients >0.7, we considered our calculated productivity to sufficiently represent Chl *a* and particulate organic carbon variations; however, without representing an actual measurement of Chl *a* or particulate organic carbon. Productivity values were then extracted for the respective 25 km^2^ pixel of each multibox corer station.

NetCDF files containing monthly means of volume, average area, and probability of finding small icebergs was downloaded from the ALTIBERG v2.1 database^64,65^. We used the raster package for R^57^ to extract data on average area of small icebergs in ha.

Summer SAM index, sea-ice cover, productivity and iceberg area data for the KNA area were used to calculate average values for each factor for, whenever possible, the 1986–2014 period. Based on these averages, we calculated summer SAM index, sea-ice cover, productivity, and iceberg area anomalies for each year.

### Statistical analysis

Abundance, biomass and complexity data were averaged for the 1988 to 2014 period. Abundance and biomass averages for each expedition were used to calculate the anomalies for the whole community and for each feeding guild. Based on the gains/losses of total benthic biomass (sum of anomalies) we extrapolated losses recorded in KNA to other Antarctic whitening shelves. We estimated the area of Antarctic whitening shelves to be 1.85 × 10^6^ km^2^. This area was calculated with the measure tool of ArcGIS 10.x and using the IBCSO chart^68^ as spatial reference.

Spearman correlation coefficients were calculated with 9999 permutations to test the association between productivity and iceberg area with benthic total abundance and biomass, for the total community and the feeding guilds. The same approach was used for 3D complexity. Negative coefficients imply a reduction (reduced), positive coefficients an increase of benthic abundance and biomass (increased) in response to the respective environmental driver investigated.

Statistical tests and figures were done using the R^57^ and ArcGIS 10.x (ESRI) softwares. R packages included for treatment and analysis of data, as well as to design figures were: raster, car, rcompanion, ggplot2, corrplot, grid, dplyr, ade4, wPerm and extrafont.

### Reporting summary

Further information on research design is available in the Nature Research Reporting Summary linked to this article.

## Supplementary information

Supplementary Information

Peer Review File

Reporting Summary

## Data Availability

The abundance and biomass data, as well as imagery transects that supports the findings of this study are made available in PANGAEA (see Supplementary Tables 1 and 3 and citations therein). Source data for Figs. 2, 3, 4, 5, Supplementary Figs. 1 and 2 are provided as Source data.

## Source data

Source Data

## References

[1] Arntz WE, Brey T, Gallardo VA (1994). Antarctic zoobenthos. Oceanogr. Mar. Biol..

[2] Gerdes D (1992). Quantitative investigations on macrobenthos communities of the southeastern Weddell Sea shelf based on multibox corer samples. Polar Biol..

[3] Gutt J, Starmans A (1998). Structure and biodiversity of megabenthos in the Weddell and Lazarev Seas (Antarctica): ecological role of physical parameters and biological interactions. Polar Biol..

[4] Gutt J, Sirenko BI, Smirnov IS, Arntz WE (2004). How many macrozoobenthic species might inhabit the Antarctic shelf?. Antarct. Sci..

[5] Gutt J, Griffiths HJ, Jones CD (2013). Circumpolar overview and spatial heterogeneity of Antarctic macrobenthic communities. Mar. Biodiv..

[6] Clarke A (1988). Seasonality in the Antarctic marine environment. Comp. Biochem. Physiol..

[7] Peck L (2005). Prospects for survival in the Southern Ocean: vulnerability of benthic species to temperature change. Antarct. Sci..

[8] Pörtner HO, Peck L, Somero G (2007). Thermal limits and adaptation in marine Antarctic ectotherms: an integrative view. Philos. Trans. R. Soc. B..

[9] Barnes DKA, Clarke A (2011). Antarctic marine biology. Curr. Biol..

[10] Barnes DKA, Clarke A (1995). Feeding activity in Antarctic suspension feeders. Polar Biol..

[11] Sumida PYG, Smith CR, Bernardino AF, Polito PS, Vieira DR (2014). Seasonal dynamics of megafauna on the deep West Antarctic Peninsula shelf in response to variable phytodetrital influx. R. Soc. Open. Sci..

[12] Gutt J (2001). On the direct impact of ice on marine benthic communities, a review. Polar Biol..

[13] Gutt J, Piepenburg D (2003). Scale-dependent impact on diversity of Antarctic benthos caused by grounding of icebergs. Mar. Ecol. Prog. Ser..

[14] Peck, L. in *Oceanography And Marine Biology: An Annual Review*, Vol. 56 (eds Hawkins, S. J., Evans, A. J., Dale, A. C., Firth L. B. & Smith, I. P.) 105–236 (CRC Press, Boca Raton, 2018).

[15] Dayton PK (1989). Interdecadal variation in an Antarctic sponge and its predators from oceanographic climate shifts. Science.

[16] Barnes DKA, Souster T (2011). Reduced survival of Antarctic benthos linked to climate-induced iceberg scouring. Nat. Clim. Change.

[17] Fillinger L, Janussen D, Lundälv T, Richter C (2013). Rapid glass sponge expansion after climate-induced Antarctic ice shelf collapse. Curr. Biol..

[18] Barnes DKA (2015). Antarctic sea ice losses drive gains in benthic carbon drawdown. Curr. Biol..

[19] Sahade R (2015). Climate change and glacier retreat drive shifts in an Antarctic benthic ecosystem. Sci. Adv..

[20] Barnes DKA, Fleming A, Sands CJ, Quartino ML, Deregibus D (2018). Icebergs, sea ice, blue carbon and Antarctic climate feedbacks. Philos. Trans. R. Soc. A..

[21] Peck LS, Barnes DKA, Cook AJ, Fleming AH, Clarke A (2010). Negative feedback in the cold: ice retreat produces new carbon sinks in Antarctica. Glob. Change Biol..

[22] Maksym T (2019). Arctic and Antarctic Sea ice change: contrasts, commonalities, and causes. Annu. Rev. Mar. Sci..

[23] Parkinson CL (2019). A 40-y record reveals gradual Antarctic sea ice increases followed by decreases at rates far exceeding the rates seen in the Arctic. Proc. Natl Acad. Sci. USA.

[24] Ludescher J, Yuan N, Bunde A (2019). Detecting the statistical significance of the trends in the Antarctic sea ice extent: an indication for a turning point. Clim. Dyn..

[25] Dayton PK (2019). Benthic responses to an Antarctic regime shift: food particle size and recruitment biology. Ecol. Appl..

[26] Dayton PK (2013). Recruitment, growth and mortality of an Antarctic hexactinellid sponge, *Anaxycalyx joubini*. Plos ONE.

[27] Dayton PK (2016). Surprising episodic recruitment and growth of Antarctic sponges: implications for ecological resilience. J. Exp. Mar. Biol. Ecol..

[28] Turner J, Hosking JS, Marsahll GJ, Phillips T, Bracegirdle TJ (2016). Antarctic sea ice increase consistent with intrinsic variability of the Amundsen Sea Low. Clim. Dyn..

[29] Comiso JC (2017). Positive trend in the Antarctic sea-ice cover and associated changes in surface tempreature. J. Clim..

[30] Vernet, M. et al. The Weddell Gyre, Southern Ocean: Present knowledge and future challenges. *Rev. Geophys*. 10.1029/2018RG000604 (2019).

[31] Ranckow T (2017). A simulation of small to giant Antarctic iceberg evolution: differential impact on climatology estimates. J. Geophys. Res. Oceans.

[32] Arrigo KR, van Dijken GL, Strong AL (2015). Environmental controls of marine productivity hot spots around Antarctica. J. Geophys. Res. Oceans.

[33] Smith CR, Minks S, DeMaster DJ (2006). A synthesis of bentho-pelagic coupling on the Antarctic shelf: food banks, ecosystem inertia and global climate change. Deep-Sea Res. Pt. II.

[34] Gutt J, Starmans A (2001). Quantification of iceberg impact and benthic recolonization patterns in the Weddell Sea (Antarctica). Polar Biol..

[35] Liu J, Curry JA, Martinson DG (2004). Interpretation of recent Antarctic sea ice variability. Geophys. Res. Lett..

[36] Abram, N. J. et al. Evolution of the Southern Annular Mode during the past millennium. *Nat. Clim. Change*. 10.1038/NCLIMATE2235 (2014).

[37] Ferreira D, Marshall J, Bitz CM, Solomon S, Plumb A (2016). Antarctic Ocean and sea ice response to ozone depletion: a two-time-scale problem. J. Clim..

[38] Doddridge EW, Marshall J (2017). Modulation of the seasonal cycle of Antarctic sea ice extent related to the southern Annular Mode. Geophys. Res. Lett..

[39] Brey T, Clarke A (1993). Population dynamics of marine benthic invertebrates in Antarctic and subantarctic environments: are there unique adaptations?. Antarct. Sci..

[40] Pearse JS, McClintock JB, Bosch I (1991). Reproduction of Antarctic benthic marine invertebrates: tempos, modes, and timing. Am. Zool..

[41] Peck L (2002). Ecophysiology of Antarctic marine ectotherms: limits to life. Polar Biol..

[42] Barnes DKA (2016). Iceberg killing fields limit huge potential for benthic blue carbon in Antarctic shallows. Glob. Change Biol..

[43] Pineda-Metz SEA, Gerdes D, Isla E (2019). Benthic communities of the Filchner Region (Weddell Sea). Mar. Ecol. Prog. Ser..

[44] Timmermann R, Hellmer HH (2013). Southern Ocean warming and increased ice shelf basal melting in the twenty-first and twenty-second centuries based on coupled ice-ocean finite-element modelling. Ocean Dynam..

[45] Sandersfeld T, Davison W, Lamare MD, Rainer K, Richter C (2015). Elevated temperature causes metabolic trade-offs at the whole-organism level in the Antarctic fish *Trematomus bernacchii*. J. Exp. Biol..

[46] Isla E, Gerdes D (2019). Ongoing ocean warming threatens the rich and diverse microbenthic communities of the Antarctic continental shelf. Prog. Oceanogr..

[47] Hellmer HH, Kauker F, Timmermann R, Hattermann T (2017). The fate of the southern Weddell Sea continental shelf in a warming climate. J. Clim..

[48] Rintoul SR (2018). Choosing the future of Antarctica. Nature.

[49] Hellmer HH, Kauker F, Timmermann R, Determan J, Rae J (2012). Twenty-first-century warming of a large Antarctic ice-shelf cavity by a redirected coastal current. Nature.

[50] Gerdes D, Hilbig B, Montiel A (2003). Impact of iceberg scouring on macrobenthic communities in the high-Antarctic Weddell Sea. Polar. Biol..

[51] Isla E (2009). Downward particle fluxes, wind and a phytoplankton bloom over a polar continental shelf: a stormy impulse for the biological pump. Mar. Geol..

[52] Alfred-Wegener-Institut Helmholtz-Zentrum für Polar- und Meeresforschung (AWI). Polar Research and Supply Vessel POLARSTERN operated by the Alfred Wegener Institute. *J. Large-scale Res. Facil*. **3**, A119 (2017).

[53] Gerdes D, Isla E, Knust R, Mintenbeck K, Rossi S (2008). Response of Antarctic benthic communities to disturbance: first results from the artificial Benthic Disturbance Experiment on the eastern Weddell Sea Shelf, Antarctica. Polar Biol..

[54] Gerdes D (1990). Antarctic trials of the multi-box corer, a new device for benthos sampling. Polar Rec..

[55] Pineda-Metz SEA, Gerdes D (2018). Seabed images versus corer sampling: a comparison of two quantitative approaches for the analysis of marine benthic communities in the southern Weddell Sea (Southern Ocean). Polar Biol..

[56] Gili JM, Coma R, Orejas C, López-González J, Zabala M (2001). Are Antarctic suspension-feeding communities different from those elsewhere in the world?. Polar Biol..

[57] R Core Team. *R: A Language and Environment for Statistical Computing* (R Foundation for Statistical Computing, 2019).

[58] Fetterer, F., Knowles, K., Meier, W., Savoie, M. & Windnagel, A. K. Sea Ice Index v3 (National Snow and Ice Data Center, 2017).

[59] NASA Goddard Space Flight Center, Ocean Ecology Laboratory & Ocean Biology Processing Group. *Sea-Viewing Wide Field-of-View Sensor (SeaWiFS) Chlorophyll Data* (NASA OB.DAAC, Greenbelt, 2018).

[60] NASA Goddard Space Flight Center, Ocean Ecology Laboratory & Ocean Biology Processing Group. *Sea-Viewing Wide Field-of-View Sensor (SeaWiFS) Particulate Organic Carbon Data* (NASA OB.DAAC, Greenbelt, 2018).

[61] NASA Goddard Space Flight Center, Ocean Ecology Laboratory & Ocean Biology Processing Group. *Moderate-Resolution Imaging Spectroradiometer (MODIS) Aqua Chlorophyll Data* (NASA OB.DAAC, Greenbelt, 2018).

[62] NASA Goddard Space Flight Center, Ocean Ecology Laboratory & Ocean Biology Processing Group. *Moderate-Resolution Imaging Spectroradiometer (MODIS) Aqua Particulate Organic Carbon Data* (NASA OB.DAAC, Greenbelt, 2018).

[63] National Oceanic & Atmospheric Administration (NOAA). *Solar Geometry Calculator*, https://www.esrl.noaa.gov/gmd/grad/antuv/SolarCalc.jsp?mu=on&sza=on&el=on&az=on (2018).

[64] Tournadre J, Bouhier N, Girard-Ardhuin F, Rémy F (2016). Antarctic icebergs distributions 1992-2014. J. Geophys. Res. Oceans.

[65] Tournadre, J., Accensi, M. & Girard-Ardhuin F. The ALTIBERG iceberg data base version-2.1 (Ifremer, 2019).

[66] Marshall, G. & National Center for Atmospheric Research Staff. *The Climate Data Guide: Marshall Southern Annular Mode (SAM) Index (Station-based)*, https://climatedataguide.ucar.edu/climate-data/marshall-southern-annular-mode-sam-index-station-based (2018).

[67] Arrigo KR, van Dijken GL, Bushinsky S (2008). Primary production in the Southern Ocean, 1997–2006. J. Geophys. Res..

[68] Arndt JE (2013). The international Bathymetric Chart of the Southern Ocean (IBCSO) Version 1.0 – a new bathymetric compilation covering circum-Antarctic water. Geophys. Res. Lett..

[69] Hasley LG (2019). The reign of the p-value is over: what alternative analyses could we employ to fill the power vacuum?. Biol. Lett..

