# Author Correction: Benthic fauna declined on a whitening Antarctic continental shelf

**DOI:** 10.1038/s41467-020-17152-1

**Published:** 2020-07-07

**Authors:** Santiago E. A. Pineda-Metz, Dieter Gerdes, Claudio Richter

**Affiliations:** 1 Alfred-Wegener-Institut Helmholtz-Zentrum für Polar- und Meeresforschung, 27568 Bremerhaven, Germany; 2 Universität Bremen (Fachbereich 2, Biologie/Chemie), 28334 Bremen, Germany

**Keywords:** Community ecology, Zoology

Correction to: *Nature Communications* 10.1038/s41467-020-16093-z, published online 06 May 2020.

The original version of this article contained an error in the first paragraph of the Discussion section, which incorrectly stated ‘If corroborated by geochemical evidence, the extrapolation of our findings to the total Antarctic continental shelf would imply an overall decrease in Antarctic zoobenthic blue carbon storage of 31.3 × 10^6^ t km^−2^ during that period, contrasting the situation described for the West Antarctic and Antarctic Peninsula regions where blue carbon has been increasing in response to climate change’. The correct version reads ‘If corroborated by geochemical evidence, the extrapolation of our findings to the total Antarctic continental whitening shelf would imply an overall decrease in Antarctic zoobenthic blue carbon storage of 18.9 × 10^6^ t during that period, contrasting the situation described for the West Antarctic and Antarctic Peninsula regions where blue carbon has been increasing in response to climate change’. This has been corrected in the PDF and HTML versions of the article.

