# Supplementary material for: Benthic fauna declined on a whitening Antarctic continental shelf
Source: Nat Commun. 2020 May 6;11:2226. doi: 10.1038/s41467-020-16093-z (PMC7203119; doi:10.1038/s41467-020-16093-z)
Supplement: Supplementary file 1 — Supplementary Information [file 41467_2020_16093_MOESM1_ESM.pdf]

Supplementary information for

BENTHIC FAUNA DECLINED ON A WHITENING ANTARCTIC CONTINENTAL SHELF

Santiago E.A. Pineda Metz[1,2*], Dieter Gerdes[1], Claudio Richter[1,2]

[1]Alfred-Wegener-Institut Helmholtz-Zentrum für Polar- und Meeresforschung, 27568 Bremerhaven, Germany

[2]Universität Bremen (Fachbereich 2, Biologie/Chemie), 28334 Bremen, Germany

*corresponding author: +49 (471) 4831 – 1706, santiago.pineda.metz@awi.de

+49 (471) 4831 – 1304, Claudio.Richter@awi.de

Supplementary Figures and captions

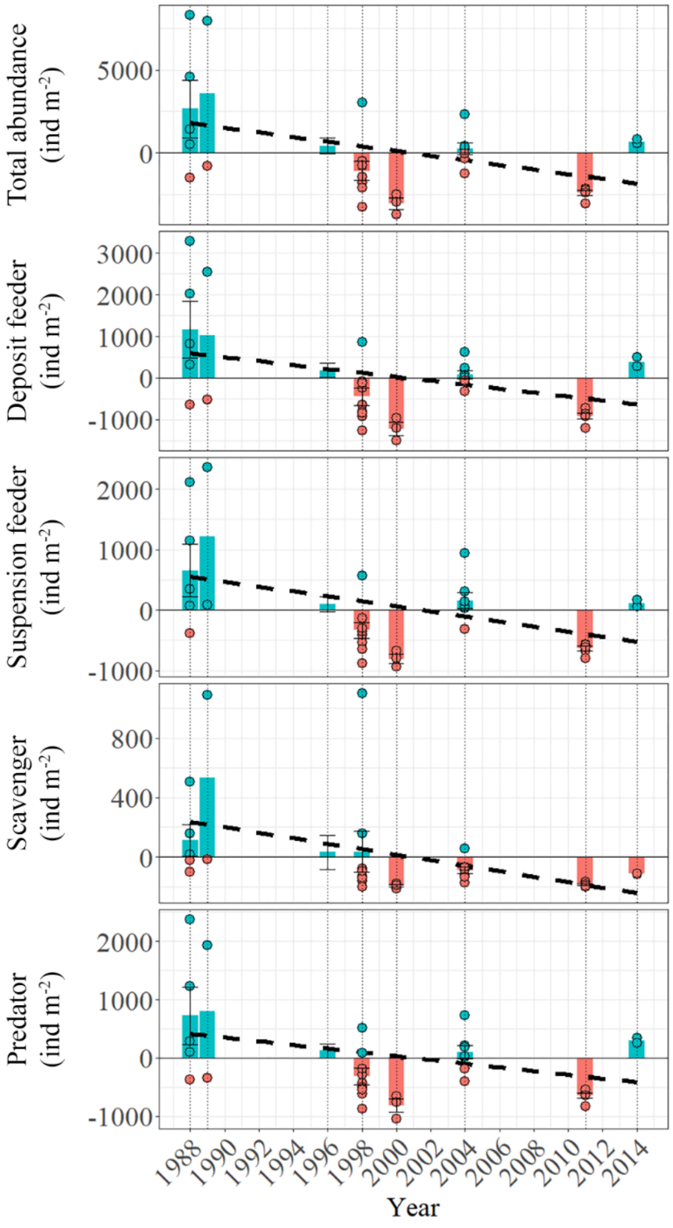

Supplementary Fig. 1. Temporal changes in benthic abundance, for (a) total community, (b) deposit feeders, (c) predators, (d) scavengers and (e) suspension feeders for the time period 1988-2014. Dashed lines represent significant Pearson linear regressions ($p < 0.05$, permutations = 9999). Dots represent independent multibox corer deployments for 1988 (n=5), 1989 (n=2), 1996 (n=24), 1998 (n=9), 2000 (n=3), 2004 (n=8), 2011 (n=6) and 2014 (n=2); note that only n≤10 are displayed. Bars represent mean values, black whiskers represent standard errors. For abundance and biomass the shading denotes n (stronger colours represent larger n). Source data can be found in the Supplementary Data: Source data

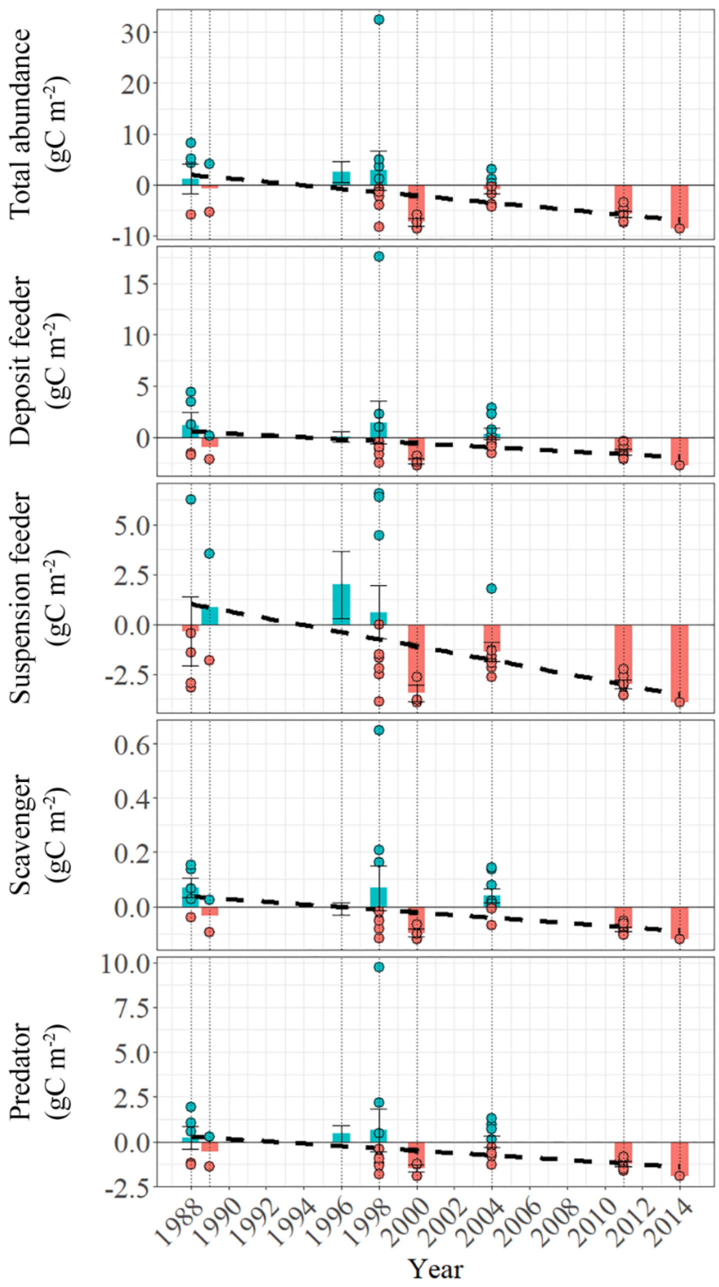

.

Supplementary Fig. 2. Temporal changes in benthic biomass, for (a) the total community, (b) deposit feeders, (c) predators, (d) scavengers and (e) suspension feeders for the time period 1988-2014. Dashed lines represent significant Pearson linear regressions ($p < 0.05$, permutations = 9999). Dots represent independent multibox corer deployments for 1988 (n=5), 1989 (n=2), 1996 (n=24), 1998 (n=9), 2000 (n=3), 2004 (n=8), 2011 (n=6) and 2014 (n=2); note that only n≤10 are displayed. Bars represent mean values, black whiskers represent standard errors. For abundance and biomass the shading denotes n (stronger colours represent larger n). Source data can be found in the Supplementary Data: Source data.

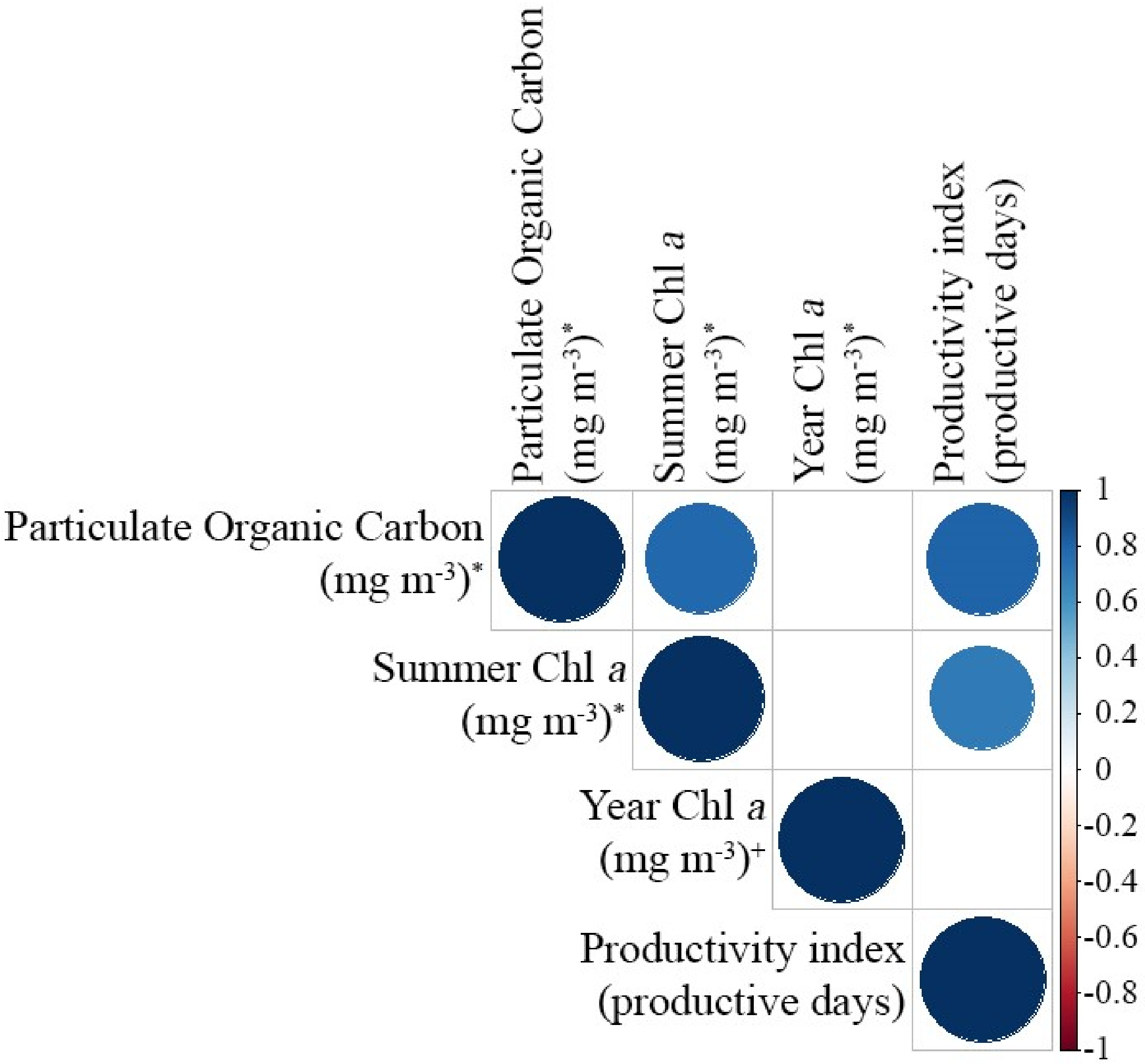

Supplementary Figure 3. Correlation matrix for Particulate Organic Carbon, Summer and Yearly Chl *a* content, and our productivity index. Asterisk (*) represents sea-surface values for the Kapp Noregia/Austasen region, data were extracted from NASA's Ocean Color data repository[1-4]. Addition symbol (+) represents Chl *a* values for the whole Weddell Sea, data were extracted from Arrigo et al.[5].

Supplementary Tables and legends

Supplementary Table 1. List of stations per expedition and year considered in our study.

Detailed abundance and biomass data for each station can be found in PANGAEA[6,7].

Station Number	Campaign (Year)	°Latitude	°Longitude	Water depth (m)	Number of cores
308	ANT-VI/3 (1988)	-71.2333	-12.9833	190	6
387		-71.3833	-13.95	308	3
418		-71.3167	-12.4167	181	8
437		-70.9667	-11.2	350	7
512		-70.7833	-10.55	266	4
274	ANT-VII/4 (1989)	-71.6183	-12.1817	211	7
277		-71.6633	-12.5817	405	7
1	ANT-XIII/3 (1996)	-71.3033	-12.2667	246	8
4		-71.3033	-12.27	174	1
5		-71.3033	-12.2717	172	2
6		-71.575	-12.4333	169	1
8		-71.5317	-13.515	574	6
9		-71.5333	-13.5183	234	6
10		-71.5367	-13.52	235	6
11		-71.51	-13.47	239	3
12		-71.6867	-12.5133	225	6
20		-71.6783	-12.76	438	5
22		-71.6683	-12.7867	224	1
23		-71.66	-12.7583	216	6
24		-71.135	-11.535	223	4
25		-71.135	-11.5317	119	1
26		-71.3283	-12.4133	118	5
27		-71.3183	-12.38	182	6
28		-71.31	-12.4233	159	5
29		-71.32	-12.45	181	5
31		-71.4867	-14.2817	628	3
33		-71.5283	-13.6367	218	3
35		-71.5317	-13.52	279	6
36		-71.5317	-13.52	241	6
37		-71.5317	-13.5167	238	4
38		-71.53	-13.52	234	3
47	ANT-XV/3 (1998)	-70.8683	-10.49	234	7
48		-70.8683	-10.4883	245	5
63		-70.8683	-10.54	234	5
67		-70.8317	-10.6083	305	2
68		-70.8367	-10.62	269	7
225		-70.085	-10.5867	276	5
227		-70.8233	-10.645	360	2
228		-70.83	-10.6333	293	4

230		-70.8467	-10.5367	229	7
90-1	ANT-XVII/3 (2000)	-71.2093	-12.6627	365	-*
120-1		-70.8383	-10.5833	271	-*
136-6		-70.8367	-10.575	256	-*
105	ANT-XXI/2 (2004)	-70.9417	-10.5335	295	6
106		-70.944	-10.5338	304	8
116		-70.9468	-10.5478	321	6
124		-70.94	-10.529	290	6
125		-70.94	-10.526	282	6
185		-70.9435	-10.5275	294	6
197		-70.9382	-10.5053	253	6
201		-70.9375	-10.5502	322	4
274	ANT-XXVII/3 (2011)	-70.9428	-10.5712	333	7
275		-70.9403	-10.527	283	8
279		-70.937	-10.5055	250	7
283		-70.966	-10.5055	284	7
295		-70.9438	-10.5335	303	5
297		-70.9433	-10.527	276	7
359	PS82 (2014)	-70.9445	-10.5372	322	8
360		-70.9418	-10.5295	283	7

Supplementary Table 2: Feeding guild assignment for all considered taxonomic units at class level. Modified after Pinda-Metz et al.[8].

	Deposit Feeders	Suspension Feeders	Scavengers	Predators
Hydrozoa	0	1	0	0
Anthozoa	0	0.13	0.0	0.87
Bryozoa	0	1	0	0
Brachiopoda	0	1	0	0
Sipuncula	1	0	0	0
Platyhelminthes	0	0	0.1	0.9
Nemertina	0	0	0	1
Priapulida	0	0	0.5	0.5
Polyplacophora	0	0	0.8	0.2
Solenogastres	1	0	0	0
Bivalvia	0.28	0.70	0	0.02
Gastropoda	0.5	0.05	0.05	0.4
Scaphopoda	0	0	0	1
Polychaeta	0.52	0.16	0.02	0.3
Clitellata	0.6	0	0	0.4
Echiurida	1	0	0	0
Acari	0	0	0	1
Pantopoda	0	0	0	1
Amphipoda	0.2	0.3	0.1	0.4

Cumacea	0.78	0.04	0	0.18
Harpacticoidea	0	0.7	0	0.3
Cirripedia	0	1	0	0
Isopoda	0.7	0	0	0.3
Tanaidacea	0	1	0	0
Ostracoda	0.3	0.2	0.4	0.2
Crustacea	0.3	0.4	0.1	0.2
Echinoidea	0.9	0.1	0	0
Holothuroidea	0.6	0.4	0	0
Asteroidea	0.2	0	0	0.8
Ophiuroidea	0.4	0.5	0	0.2
Crinoidea	0	1	0	0
Hemichordata	0.6	0.4	0	0
Tunicata	0	1	0	0
Unidentified	0.25	0.25	0.25	0.25

Supplementary Table 3. List of imagery transects per expedition and year considered in the benthic 3D complexity analysis.

Station	Campaign (Year)	°Start Latitude	°Start Longitude	Average water depth (m)	Type of imagery transect	Reference
305-1	ANT-VI/3 (1988)	-71.12	-12.99	454	Video	Gutt & Klindt[9]
307-1		-71.14	-12.30	375		
308-1		-71.25	-12.99	173		
308-2		-71.25	-12.99	175		
387-1		-71.38	-13.96	264		
387-2		-71.32	-12.41	159		
418-1		-71.32	-12.41	160		
418-2		-70.96	-11.10	293		
424-1		-70.85	-10.99	351		
438-1		-70.85	-10.99	330		
438-2		-70.79	-10.56	282		
512-1		-71.12	-12.99	454		
005-05	ANT-XIII/3 (1996)	-71,68	-12,74	233	Photograph	Gutt[10]
005-10		-71,67	-12,73	236		
006-01		-71,52	-13,51	225		
006-09		-71,53	-13,52	247		
006-18		-71,53	-13,45	222		
006-22		-71,53	-13,52	246		
007-01		-71,45	-13,72	212		
007-07		-71,44	-13,73	215		
008-03		-71,30	-12,26	175		
008-11		-71,28	-12,25	172		
009-03		-71,57	-12,44	599		
009-08		-71,44	-13,71	214		

023-01		-71,96	-15,84	349		
024-01		-71,12	-11,51	146		
024-03		-71,12	-11,45	140		
024-09		-71,11	-11,53	165		
024-12		-71,10	-11,57	189		
025-15		-71,38	-14,32	649		
026-01		-71,53	-14,20	226		
026-02		-71,58	-14,13	213		
026-03		-71,49	-14,27	200		
026-08		-71,48	-14,31	216		
027-01		-71,34	-12,40	195		
032-01		-70,51	-8,51	182		
032-02		-70,52	-8,50	145		
032-05		-70,49	-8,66	190		
032-06		-70,54	-8,60	138		
40	ANT-XV/3 (1998)	-70,87	-10,52	235	Photograph	Gutt & Teixidó[11]
41		-70,90	-10,56	247		
45		-70,87	-10,47	247		
52		-70,87	-10,48	247		
59		-70,89	-10,46	238		
65		-70,87	-10,54	229		
66		-70,85	-10,51	234		
70		-70,82	-10,48	282		
72		-70,85	-10,52	231		
73		-70,83	-10,51	288		
74		-70,82	-10,48	281		
185		-71,53	-14,38	169		
186		-71,53	-14,40	169		
190		-71,69	-12,75	238		
192		-71,23	-12,42	255		
193		-71,23	-12,43	253		
200		-71,26	-13,15	162		
201		-71,27	-13,18	163		
213		-71,13	-11,47	114		
215		-71,11	-11,53	163		
219		-70,84	-10,55	267		
221		-70,84	-10,59	278		
226		-70,84	-10,58	269		
229		-70,85	-10,52	236		
232		-70,82	-10,48	284		
238		-71,10	-11,53	205		
239		-71,11	-11,53	208		
242		-71,27	-12,33	166		
278		-70,89	-10,70	293		
281		-70,67	-8,03	68		
285		-70,79	-10,41	296		
286		-70,86	-10,75	318		

287		-71,23	-12,25	277		
081-1	ANT-XVII/3 (2000)	-71,21	-12,35	315	Photograph	Gutt et al.[12]
082-1		-71,25	-12,54	375		
083-1		-71,25	-12,49	328		
084-1		-71,24	-12,46	253		
094-1		-71,12	-12,65	446		
107-1		-71,15	-12,54	396		
125-1		-70,77	-10,57	322		
126-1		-70,83	-10,58	277		
127-1		-70,87	-10,52	246		
091-1	ANT-XXI/2 (2004)	-70,93	-10,50	233	Video	Gutt et al.[13]
102-1A		-70,94	-10,53	285		
102-1B		-70,94	-10,54	303		
184-1B		-70,94	-10,51	252		
184-1C		-70,94	-10,50	238		
250-1		-71,13	-11,50	229		
227-1A		-71,12	-11,45	238		
227-1B		-71,12	-11,47	245		
227-1C		-71,12	-11,50	259		
335-1A		-70,84	-10,46	272		
335-1B		-70,85	-10,48	246		
264-1	ANT-XXVII/3 (2011)	278	-70,95	-10,52	Video	*
278-1		206	-70,91	-10,32		
287-1		178	-70,98	-10,45		
352	PS82 (2014)	-70.93	-10.54	291	Video	Owsianowski et al.[14]
359		-70.94	-10.54	322	Photograph	Pineda-Metz & Gerdes[15]
360		-70.94	-10.53	283		

*To be published in PANGAEA

Supplementary Method "Productivity Raster calculation"

As primary data we used the daily sea-ice cover data for the period 1986-2014 provided as raster by the National Sea Ice Data Center (NSIDC)[1]. Additional to this, the Solar Geometry Calculator of the National Oceanic and Atmospheric Administration (NOAA)[2] was used to calculate the average proportion of a summer day with sun light during for the period 1986-2014. Here is a step by step explanation of the processing of these data to compute

1. All daily sea-ice cover raster for the whole Southern Ocean were stacked by month and year. Stacks were named by the first letter of the month (first two when needed, e.g. April and August) and last two digits of the year. E.g. all raster corresponding to January 2000 were stacked into j00, whereas all raster of August 1999 were stacked into au99.
2. All stacks were further grouped by summer and named as s_i, where i represents the last two digits of the latest year. E.g. the stack s00 (summer 2000) is composed of n99, d99, j00, f00 and m00.
3. Sea-ice cover data of all cells of a given summer stack were transformed to 0 or 1 values, where 1 corresponds to ice-free day and 0 to ice-covered day. We defined ice-free day as a day with sea-ice cover < 15%. These stacks were renamed as ifd_i, where i represents the year. E.g. ifd00 correspond to the transformed data for the summer of 2000.
4. Since the data set showed gaps, i.e. data for some days was missing or not recorded, we first calculated a raster consisting of the proportion of ice-free days in a given ifd_i stack. This was done with the following formula:

$$pifd_i = (sum(ifd_i)/nlayer(ifd_i))$$

   Where "sum(ifd_i)" is the total amount of ice-free days for each cell in any ifd_i stack, and "nlayers(ifd_i)" is the number of layers in the stack, which represents the amount of days with data.

   E.g. The stack ifd_{86}, which represents summer data for 1986, has only 75 days with data, thus the raster $pifd_{86}$ would be calculated as

$$pifd_{86} = (sum(ifd_{86})/75)$$

   - The proportion calculated for each cell was then multiplied by the amount of summer days of a non-leap year (151 days) in the final productivity calculation.
5. Once all $pifd_i$ raster were calculated, the following formula was used to calculate productivity for all cells of any given $pifd_i$ raster:

$$Productivity = (pifd_i * 151) * pl$$

   Where "pl" is a constant representing the average proportion of a summer day with sun light (0.8762). Raster were named p_i, where i corresponds the summer year. E.g. p00 corresponds to the productivity raster calculated form the stack s00.

6. After all productivity raster were calculated, data for all cells within the Kapp Norvegia/Auståsen (KNA) polygon was extracted in the GIS environment and used to calculate productivity in KNA for any given year within the 1986-2014 period.

The following code is an extraction of the R code used to calculate all productivity raster, and corresponds to the summer of 2000:

library(raster)

setwd("n") <- n corresponds to the pathway to the folder where all daily sea-ice cover raster files are located

\#	To create stacks for each month per year

```
n99<-stack(list.files(pattern="n_99"))

d99<-stack(list.files(pattern="d_99"))

j00<-stack(list.files(pattern="j_00"))

f00<-stack(list.files(pattern="f_00"))

m00<-stack(list.files(pattern="m_00"))

#	To create summer stack

s00<-stack(n99,d99,j00,f00,m00)

#	To transform sea-ice cover data into ice-free / ice-covered day data

“NOTE: NSIDC sea-ice cover data ranges from 0 to 1000, where 1000 represents 100%, and 10
represents 1%. Furthermore, these raster include data with values over 1000 representing land mass
and missing values.”

ifd00<-s00

ifd00[ifd00>1000]<-NA

ifd00[ifd00<150]<-1

ifd00[ifd00>150]<-0

#	To calculate pifd raster

pifd00<-sum((s00)/nlayers(s00)

#	To calculate productivity raster

p00<-(pifd00*151)*0.8762

#	To save the raster as asci file

writeRaster(pa,filename = "p00.asc",format="ascii")
```

SUPPLEMENTAL LITERATURE

1- NASA Goddard Space Flight Center, Ocean Ecology Laboratory, Ocean Biology Processing Group. Sea-viewing Wide Field-of-view Sensor (SeaWiFS) Chlorophyll Data. NASA OB.DAAC, Greenbelt, MD, USA. doi:data.10.5067/ORBVIEW-2/SEAWIFS/L3B/CHL/2018 (2018a).

2- NASA Goddard Space Flight Center, Ocean Ecology Laboratory, Ocean Biology Processing Group. Sea-viewing Wide Field-of-view Sensor (SeaWiFS) Particulate Organic Carbon Data. NASA OB.DAAC, Greenbelt, MD, USA. doi:data.10.5067/ORBVIEW-2/SEAWIFS/L3B/POC/2018 (2018b).

3- NASA Goddard Space Flight Center, Ocean Ecology Laboratory, Ocean Biology Processing Group. Moderate-resolution Imaging Spectroradiometer (MODIS) Aqua Chlorophyll Data. NASA OB.DAAC, Greenbelt, MD, USA. doi:data.10.5067/AQUA/MODIS/L3B/CHL/2018 (2018c).

4- NASA Goddard Space Flight Center, Ocean Ecology Laboratory, Ocean Biology Processing Group. Moderate-resolution Imaging Spectroradiometer (MODIS) Aqua Particulate Organic Carbon Data. NASA OB.DAAC, Greenbelt, MD, USA. doi:data.10.5067/AQUA/MODIS/L3B/POC/2018 (2018d).

5- Arrigo, K.R., van Dijken, G.L. & Bushinsky, S. Primary production in the Southern Ocean, 1997–2006. *J. Geophys. Res.* **113**, C08004. doi:10.1029/2007JC004551 (2008).

6- Pineda-Metz, S.E.A. & Gerdes, D. Abundance of macrozoobenthos in surface sediments sampled with a multiboxcorer between 1988 and 2014. PANGAEA https://doi.pangaea.de/10.1594/PANGAEA.913980 (2020)

7- Pineda-Metz, S.E.A. & Gerdes, D. Biomass (in gC/m²) of macrozoobenthos in surface sediments sampled with a multiboxcorer between 1988 and 2014. PANGAEA https://doi.pangaea.de/10.1594/PANGAEA.913619 (2020)

8- Pineda-Metz, S.E.A., Gerdes, D. & Isla, E. Benthic communities of the Filchner Region (Weddell Sea). *Mar. Ecol. Prog. Ser.* **628**, 37-54. doi:10.3354/meps13093 (2019).

9- Gutt, J. & Klindt, H. Sea-floor videos and photographs (benthos) along 16 ROV profiles during POLARSTERN cruise ANT-VI/3, Weddell Sea. PANGAEA https://doi.org/10.1594/PANGAEA.757740 (2011).

10- Gutt, J. Seafloor images during POLARSTERN cruise ANT-XIII/3 to the Weddell Sea, Antarctica. PANGAEA https://doi.org/10.1594/PANGAEA.755488 (2010).

11- Gutt, J. & Teixidó, N. Sea-floor images from during POLARSTERN cruise ANT-XV/3 to the Weddell Sea, Antarctica. PANGAEA https://doi.org/10.1594/PANGAEA.755491 (2004).

12- Gutt, J., Starmans, A. & Teixidó, N. Sea-floor images from ROV transects during POLARSTERN cruise ANT-XVII/3 (EASIZ III) to the Weddell Sea, Antarctica. PANGAEA https://doi.org/10.1594/PANGAEA.755490 (2010).

13- Gutt, J. et al. Sea-floor videos and photographs (bentos) along 6 shelf profiles from the eastern Weddell Sea, Antarctica taken with remote operated vehicle CHEROKEE during POLARSTERN cruise ANT-XXI/2. PANGAEA https://doi.org/10.1594/PANGAEA.770359 (2011).

14- Owsianowski, N., Federwisch, L., Kluibenschedl, A., Casado de Amezua, M. & Richter, C. Sea-floor videos (benthos) along 12 ROV profiles during POLRASTERN cruise PS82 (ANT-XXIX/9). PANGAEA

https://doi.org/10.1594/PANGAEA.879283 (2017).

15 - Pineda-Metz, S.E.A. & Gerdes, D. Seabed images taken during POLARSTERN cruise ANT-XXIX-9 (PS82) to the Weddell Sea, Antarctica. PANGAEA. https://doi.pangaea.de/10.1594/PANGAEA.914197 (2020)

16- Fetterer, F., Knowles, K., Meier, W., Savoie, M. & Windnagel, A.K. Sea Ice Index, Version3 [01 January 1979 to 31 December 2017]. Boulder, Colorado USA. NSIDC: National Snow and Ice Data Center. doi:10.7265/N5K072F8 (2018).

17- National Oceanic & Atmospheric Administration (NOAA). Solar Geometry Calculator. https://www.esrl.noaa.gov/gmd/grad/antuv/SolarCalc.jsp?mu=on&sza=on&el=on&az=on (2018).
