# Supplementary material for: Benthic fauna declined on a whitening Antarctic continental shelf
Source: Nat Commun. 2020 May 6;11:2226. doi: 10.1038/s41467-020-16093-z (PMC7203119; doi:10.1038/s41467-020-16093-z)
Supplement: Supplementary file 2 — Peer Review File [file 41467_2020_16093_MOESM2_ESM.pdf]

Peer Review File

Reviewers' comments:

Reviewer #1 (Remarks to the Author):

This is an extremely valuable paper. It offers unique data that will never be matched. We are very lucky that the AWI had the prescience to initiate the long-term study and that many scientists have so faithfully stayed with it. I am familiar with others such as kelps and McMurdo Sound benthos, but nothing in the world integrates the water column to the benthos over such an important spatial scale. The CalCOFI program is the only thing to match it, but they do not do the benthic community.

This paper is extremely terse as the authors tried to get so much into the paper that it is hard for me to read. This is largely my fault as I have not kept up with the blue and white carbon concerns and the jargon is new to me, but after several reads I have not found anything that concerns me. One immediate suggestion to help the more general readers is that you might be more clear in the beginning that you are looking at changes associated with increasing sea ice. Most people assume that the ice is melting and the increase in your area is not widely appreciated. This is mentioned in the abstract, and perhaps that is good enough, but you might add some of the focus on ice cover from the discussion which is very clear.

Given the focus on carbon deposition, the authors have had to skimp on the amount of interesting natural history. I found myself wondering which species were responsible for most of the changes and wondering about the processes driving the changes at the species level. I don't think that it is possible to get more into the paper given the space limits imposed by Nature, but I hope that the authors might write other more detailed papers where space is not so limiting.

My only technical suggestion is that there is an extra "Literature" in the middle of page 15. How is that for a trivial criticism?
I suppose other reviewers more knowledgeable about the topic might find issues to quibble over, but I have read it 5 times and while I do not understand all (maybe most?) of the details, I really do consider this paper to be extremely valuable and nothing will ever be done to cover this really important change in a large coastal ecosystem. It is truly unique and while it might be improved here and there, it has to be published!

Reviewer #2 (Remarks to the Author):

This manuscript investigates aspects of sea ice and benthic fauna in the Weddell Sea, Antarctica. The authors main idea was to assess various measures of benthos performance against coincident environmental change, which have been derived from various literature and database sources. They have built up an impressive faunal data set spanning nearly 30 years. They have used various methods to derive benthos performance and thought carefully about which samples and data are applicable, and for good reasons ruled some out. Their overall finding is that biomass has decreased, coincident with increased sea ice and iceberg cover. This data and report are an asset to this field as most work in this subject area has focussed on West Antarctica where, as the authors rightly point out, sea ice (and many other physical parameters) have shown v different patterns. So I think the work should be interesting to a range of readers and attention is overdue to an 'East Antarctic' shelf. Whether the Weddell sea represents other East Antarctic shelves remains to be seen – after all literature data shows faunas across West Antarctic shelves losing ice perform differently. The authors acknowledge that 'The end of our study marks the possible end of three decades of increasing Antarctic sea-ice extent with a record high in 2014 followed by a precipitous decline to a record low only three years later' in the discussion yet the title and abstract give the impression that it is still 'whitening' – which it clearly isn't. On the whole the ms is quite well written and appropriate in length. I found it particularly interesting that 3 of their 4 feeding guilds show an almost identical pattern with time (suppl fig 1) – despite such major

differences in diet and lifestyle. Overall I think that this ms should be publishable with revisions.

1) Error. This may be bigger than reported for two reasons.
a) The authors need to be clear about true replication, suppl Table 1 suggests that they took (6+3+8+7+4 = 28) replicates in 1988. In my (and most I am aware of) opinion a deployment to a spot on the seabed is a sample (=replicate), thus there was really 5 replicates in that year. In 1989 just 2 rather than 14 etc. This is important because continental shelf faunas are very patchy, and multiple graps within a single deployment are likely to fall within a patch, thus be more similar than between samples, making error appear low.
b) What was the error associated with imagery? Calculating megafaunal density from visual data requires counting within a set area. What was the size of this area, how much did it vary between image samples and how accurately could they measure this? This is important because error associated with imagery can be very considerable. A camera perpendicular to the seabed (eg on a frame) is likely to have much less error between images than a vertical one (unless the seabed is always horizontally flat). Video tends to be shot forward facing so can have huge amounts of error when calculating area (lasers help measurement in a plane but not an area). Please include a table with error calculations if this was measured or declare that it was not measured (which does not make the data unpublishable but it does alert the reader to data reliability). Recalculate true replicates for table 1.
2) Statistics. Because of replication & error issues point 1 above I think regression statistics should be recalculated (for Fig 2 and suppl fig 1), using true replicates. The biomass decrease is a very noisy signal and it would be reassuring for a reader to know that the relationship was still significant with the reduced (what I would term, genuine) replication. Suppl figs 3 & 4 each include 91 correlations, yet they use $p<0.05$ as the critical threshold. Without correction, even $p=0.01$ would give false significant correlations by chance with that many tests, I would have thought that $p<0.005$ would be a more appropriate threshold.
3) Productivity. I am not convinced about plotting productivity when it was not actually measured. It was derived from generalised relationship [across a very large area] from literature data of one of the other parameters plotted. A casual reader might think that that what they term productivity was actually measured. Accuracy of deriving information from remotely sensed data varies from place to place and just because it works reasonably well for the Southern Ocean or even Weddell sea as a whole doesn't necessarily mean it is strong for their study site. I would either delete that last (bottom) plot from Fig 2 or demonstrate that it has been ground-truthed as robust to the specific region in question. Likewise I would delete productivity measures from figure 5 unless genuine productivity data can be produced to show support for the derived estimate.
4) Iceberg scouring. As with productivity, impact frequency with the seabed was not measured or ground truthed. Their plots rely on number and area of icebergs, neither of which are strong predictors of seabed impact rate. Presence of icebergs doesn't indicate scouring, and area is not a great predictor of keel depth, is there any relevance of small icebergs in such depths (unlikely to be scouring). Look at seabed multibeam data - Scour density is often highest at shelf breaks - where there are few icebergs, but collisions are big because the seabed can be a bit shallower and icebergs gather more momentum with current and wind. Iceberg activity has been shown to decrease with 'whitening' because they can move around less [to collide with seabed]. Showing more icebergs does not imply more scouring. As with productivity it is implication rather than any direct measurement or ground trothing so I would delete this section, again not needed for the central story.

Minor points
P1 The title '..on a whitening Antarctic continental shelf.', abstract 'In much of the East Antarctic, however, sea-ice has increased over the last decades' and start of the discussion are misleading. As the authors say later that 'increase' in sea ice has since been lost, and the net loss around Antarctica is now the same as 30 years of loss around the Arctic.

P2 Remove 'The lack of crushing predators has led to a rich epifauna dominated by sessile suspension feeders'? it does not add to the argument and is contentious. There is certainly 'a rich epifauna dominated by sessile suspension feeders' around Antarctica, whether it is due to rarity of crushing predators is opinion. There are other places dominated by sessile, primary consumers (inc suspension feeders) that have duraophagus predators.
Change '..carbon captured by benthos' to '..carbon stored by benthos'.

P5 and throughout change ‘community’ to ‘assemblage’. Community refers to the foodweb with all of its components, the authors just measure part of that community – the macro and megabenthos.
- Change ‘Our results provide important ground-truthing of the proposed links between productivity, iceberg presence and benthic abundance and biomass‘ to ‘Our results provide important ground-truthing of the proposed links between sea ice and iceberg presence and benthic biomass’ (ie delete productivity and abundance [the latter since no relationship is demonstrated], but include sea ice.
- Delete ‘and refocus the attention to the larger part of the Antarctic continental shelf that has been whitening: only the Bellingshausen/Amundsen sector of the Antarctic has seen a negative trend in sea-ice extent over 40 years of satellite observations, whereas the remainder of the Antarctic has seen increasing trends in sea-ice extent27,43 so that, with the exception of the Bellingshausen/ Amundsen sector, an overall decrease in blue carbon can be expected.’ Firstly the Scotia sea consistently lost sea ice as well, but recent papers eg Turner & Comiso 2017 show virtually all sectors have now lost net sea ice over that period.
- I’m not convinced that ‘Extrapolating our findings from the KNA area (Fig. 2) to whitening Antarctic shelves we calculate a 31.3 x 106 t km-2 blue carbon loss over the study period’ is valid (even assuming that the trend stays true with recalculated replication). It is certainly a loss of ‘blue carbon storage’ but some of that may be buried in berms by icebergs. The key value of blue carbon as an ecosystem service is removal from the carbon cycle, ie burial to sequestration, which is much more ‘valuable’ than blue carbon storage in living benthos. Examining the organic carbon subsurface profile in multicores would be able to confirm whether it is actually a net loss.. or whether much of that blue carbon storage has been converted to sequestered carbon. The authors could make the sentence true by inserting the words ‘zoobenthic’ before and ‘storage’ after blue carbon but I think that would be disingenuous without the corroborating geochemistry from core profiles to support it; otherwise delete it, the ms is still okay without it.
- As a general comment, not all blue carbon is equal - if the point of evaluating it is to assess an ‘societal ecosystem service’. The authors could make their data more valuable to assessment of blue carbon by splitting their groups of fauna up more from merely feeding guilds. On death, the carbon in soft bodied forms such as an errant polychaete worm are nearly all entirely recycled in the microbial loop. In contrast carbon within heavily skeletalised forms are much more likely to sequester that carbon (hence their more obvious fossil record).

P7 The box cores sample a precise area of seabed, not so most cameras. Please include more detail on the nature and error associated with the imagery used. What angles were the camera and video to the seabed? How large was the field of view and the area of analysis within each imagery sample? Importantly, how was area within image calculated? Please provide an extra suppl table showing levels of error of area calculation within the image and video samples. The reader should be informed about data reliability [extracted from imagery].

P9 The iceberg methodology and data is weak and unsubstantiated so I would remove.

Reviewer #1:

This is an extremely valuable paper. It offers unique data that will never be matched. We are very lucky that the AWI had the prescience to initiate the long-term study and that many scientists have so faithfully stayed with it. I am familiar with others such as kelps and McMurdo Sound benthos, but nothing in the world integrates the water column to the benthos over such an important spatial scale. The CalCOFI program is the only thing to match it, but they do not do the benthic community.

This paper is extremely terse as the authors tried to get so much into the paper that it is hard for me to read. This is largely my fault as I have not kept up with the blue and white carbon concerns and the jargon is new to me, but after several reads I have not found anything that concerns me. One immediate suggestion to help the more general readers is that you might be more clear in the beginning that you are looking at changes associated with increasing sea ice. Most people assume that the ice is melting and the increase in your area is not widely appreciated. This is mentioned in the abstract, and perhaps that is good enough, but you might add some of the focus on ice cover from the discussion which is very clear.

Reply: This is an important point we took up in the abstract and introduction of the revised manuscript.

The abstract now reads (Page 1, Lines 9-11):

"Ice retreat in West Antarctica and Antarctic Peninsula has led to important changes in seafloor communities and gains in benthic blue carbon. In most of the Antarctic, however, sea ice has increased between the seventies and 2014, but its effects on the benthos remain largely unexplored."

The introduction now reads (Page 2, Lines 12-18):

"Most studies on the Antarctic benthos in a changing climate are in response to glacier, sea-ice, anchor ice retreat, increased iceberg scouring and ice-shelf collapse[15,16,17,18,19], establishing a link between pelagic productivity and carbon stored by benthos (blue carbon)[18,20,21]. As opposed to the Arctic where sea-ice has been plummeting over the last decades[22], two thirds of the Antarctic including the Weddell Sea, the East Antarctic shelf and the Ross Sea have seen increasing trends of sea-ice over the satellite instrumental period from 1979 up to a maximum in 2014[23,24], but the response of the benthos to this larger 'whitening' region of the Antarctic is so far unexplored..."

Given the focus on carbon deposition, the authors have had to skimp on the amount of interesting natural history. I found myself wondering which species were responsible for most of the changes and wondering about the processes driving the changes at the species level. I don't think that it is possible to get more into the paper given the space limits imposed by Nature, but I hope that the authors might write other more detailed papers where space is not so limiting.

Reply: A detailed account about the species and processes driving the changes would have been beyond the scope of the paper, but we are gladly taking up the suggestion to follow up on this paper with more detail elsewhere.

My only technical suggestion is that there is an extra "Literature" in the middle of page 15.

Reply: The "Literature" in the middle of page 15 is an overlooked left-over from an earlier version of the manuscript. We removed it in the revised version of the manuscript.

Reviewer #2:

This manuscript investigates aspects of sea ice and benthic fauna in the Weddell Sea, Antarctica. The authors main idea was to assess various measures of benthos performance against coincident environmental change, which have been derived from various literature and database sources. They have built up an impressive faunal data set spanning nearly 30 years. They have used various methods to derive benthos performance and thought carefully about which samples and data are applicable, and for good reasons ruled some out. Their overall finding is that biomass has decreased, coincident with increased sea ice and iceberg cover. This data and report are an asset to this field as most work in this subject area has focussed on West Antarctica where, as the authors rightly point out, sea ice (and many other physical parameters) have shown v different patterns. So I think the work should be interesting to a range of readers and attention is overdue to an 'East Antarctic' shelf.
Whether the Weddell sea represents other East Antarctic shelves remains to be seen – after all literature data shows faunas across West Antarctic shelves losing ice perform differently. The authors acknowledge that 'The end of our study marks the possible end of three decades of increasing Antarctic sea-ice extent with a record high in 2014 followed by a precipitous decline to a record low only three years later' in the discussion yet the title and abstract give the impression that it is still 'whitening' – which it clearly isn't.

Reply: Our description of a whitening Antarctic refers to the 26-year study period 1988-2014, not to the period beyond for which we have no data. However, to avoid the impression that Antarctic sea ice may still be increasing, we modified the abstract. Page 1; Lines 9-11 now reads "Ice retreat in West Antarctica and Antarctic Peninsula has led to important changes in seafloor communities and gains in benthic blue carbon. In most of the Antarctic, however, sea ice has increased between the seventies and 2014, but its effects on the benthos remain largely unexplored."

On the whole the ms is quite well written and appropriate in length. I found it particularly interesting that 3 of their 4 feeding guilds show an almost identical pattern with time (suppl fig 1) – despite such major differences in diet and lifestyle. Overall I think that this ms should be publishable with revisions.

1) Error. This may be bigger than reported for two reasons.
a) The authors need to be clear about true replication, suppl Table 1 suggests that they took (6+3+8+7+4 = 28) replicates in 1988. In my (and most I am aware of) opinion a deployment to a spot on the seabed is a sample (=replicate), thus there was really 5 replicates in that year. In 1989 just 2 rather than 14 etc. This is important because continental shelf faunas are very patchy, and multiple graps within a single deployment are likely to fall within a patch, thus be more similar than between samples, making error appear low.

Reply: The introduction of multiple sampling devices, such as the multicorer or the multibox corer, has been accompanied by questioning the independence of samples (e.g. Blomqvist 1991). The non-conservative stance has been that the distance between neighbouring cores (boxes) is larger than the autocorrelation distance for the infauna (e.g. Montagna et al. 2017); the conservative view has been that the independence of the samples is violated by the simple fact that the distance between neighbouring samples is governed by the given fixed diameter of the instrument (Blomqvist 1991). In the revised ms we adopted the conservative approach, as suggested by the reviewer, and recalculated the error by taking into account the station averages, rather than the box values. We carried out distribution-free permutation tests to detect statistical differences between years (Anderson 2001). The revised analysis supports our previous conclusion that macrofauna biomass and abundance declined over the study period. It also supports, in

most cases, the previous links between the observed changes in macrofauna feeding guilds and the changes in productivity (sea ice) and and iceberg, albeit with lower but mostly significant levels of confidence. The revised results are summarized in the updated Table 1, as well as Fig. 2 & 5, and Supp. Fig. 1 & 2 and are described in the revised text (Page 3, Line 15-Page 5, Line 16).

- Anderson, M.J. Permutation tests for univariate or multivariate analzsis of variance and regression. *Can. J. Fish. Aquat. Sci.* **58**, 626–639. doi:10.1139/cjfas-58-3-626 (2001).
- Blomqvist, S. Quantitative sampling of soft-bottom sediments: problems and solutions. *Mar. Ecol. Prog. Ser.* **72**, 295–304 (1991).
- Montagna, P.A., Baguley, J.G., Hsiang, C.-Y. & Reuscher M.G. Comparison of sampling methods for deep-sea infauna. *Limnol. Oceanogr. Methods* **15**, 166–183. doi:10.1002/lom3.10150 (2017).

b) What was the error associated with imagery? Calculating megafaunal density from visual data requires counting within a set area. What was the size of this area, how much did it vary between image samples and how accurately could they measure this? This is important because error associated with imagery can be very considerable. A camera perpendicular to the seabed (eg on a frame) is likely to have much less error between images than a vertical one (unless the seabed is always horizontally flat). Video tends to be shot forward facing so can have huge amounts of error when calculating area (lasers help measurement in a plane but not an area). Please include a table with error calculations if this was measured or declare that it was not measured (which does not make the data unpublishable but it does alert the reader to data reliability).

Reply: Megafauna was not quantified, hence we did not calculate errors or take into account reference areas or observation angles. Megafauna was described on a categorical basis using an ordinal scale ranging from 0 to 5. Category 0 represented fresh scours, defined as absence of visible fauna in a frame/image. Categories 1 to 5 represent one of the 5 successional stages of Antarctic shelf megafaunal assemblages sensu Gili et al. (2001), of increasing three-dimensionality (see Fig. 1, in Gili et al. (2001)). Terrestrial analogs are found in landscape ecology where the distinction between forest, savanna, grasslands, etc. is possible without exact knowledge of counts, areas, angles, etc.

In the revised manuscript we replaced the original text by the following (Page 7, Line 20-Page 8, Line 10):

"To assess changes in the structural complexity of the megafauna from a flat (early successional stage) to a three-dimensional mature apex community dominated by megafauna (glass sponges, gorgonians), we analysed video- and photo-transects (i.e. imagery transects) from all expeditions, with the exception of 1989 for which no image data are available. Transects representing the years 1988, 1996, 1998, 2000, 2004, and one transect for 2014 (Supplementary Table 3), were extracted from the PANGAEA data repository.

Megafauna was described on a categorical basis using an ordinal scale ranging from 0 to 5. Category 0 represented fresh scours[34], defined as absence of visible fauna in a frame/image. Categories 1 to 5 represent one of the five successional stages of Antarctic shelf megafaunal assemblages sensu Gili et al.[57], of increasing three-dimensionality (see Fig. 1, in Gili et al. [57]). The frequency of all 3D configurations was assessed for all imagery transects, and was represented by five categories (from 0 to 4) to facilitate analysis. Each category represents the following proportions: 0 = absent/rare, 1 = uncommon, 2 =

common, 3 = mainly, 4 = dominant. This first analysis gave a transect x 3D category table which was used with the "prep.fuzzy" function of the "ade4" package for R[58] to transform categorical values to percentage. These calculated percentages were used to estimate the average proportion of each 3D configuration category at each year."

- Gili, J.M., Coma, R., Orejas, C., López-González, J. & Zabala, M. Are Antarctic suspension-feeding communities different from those elsewhere in the world? *Polar Biol.* **24**, 473–485 (2001).

2) Statistics. Because of replication & error issues point 1 above I think regression statistics should be recalculated (for Fig 2 and suppl fig 1), using true replicates. The biomass decrease is a very noisy signal and it would be reassuring for a reader to know that the relationship was still significant with the reduced (what I would term, genuine) replication. Suppl figs 3 & 4 each include 91 correlations, yet they use $p<0.05$ as the critical threshold. Without correction, even $p=0.01$ would give false significant correlations by chance with that many tests, I would have thought that $p<0.005$ would be a more appropriate threshold.

Reply: We have re-calculated the regression statistics using the station averages instead of the box samples and performed a permutation analysis to test for significant temporal differences. We present the data in the revised Fig. 2 and in the text (Page 3, Lines 17-23). We agree that $p < 0.005$ is an appropriate threshold for significant correlations. We removed all supplementary figures exceeding the threshold (original Supplementary Fig. 3, 4 and 5) from the revised manuscript.

3) Productivity. I am not convinced about plotting productivity when it was not actually measured. It was derived from generalised relationship [across a very large area] from literature data of one of the other parameters plotted. A casual reader might think that that what they term productivity was actually measured. Accuracy of deriving information from remotely sensed data varies from place to place and just because it works reasonably well for the Southern Ocean or even Weddell sea as a whole doesn't necessarily mean it is strong for their study site. I would either delete that last (bottom) plot from Fig 2 or demonstrate that it has been ground-truthed as robust to the specific region in question. Likewise I would delete productivity measures from figure 5 unless genuine productivity data can be produced to show support for the derived estimate.

Reply: We acknowledge the reviewer's concern that proxies derived over a wide area may not provide reliable results on a regional or local scale. We addressed this concern by calibrating our derived 'productivity' data against Chl *a* and POC data available for our study region (NASA 2018a-d; https://oceancolor.gsfc.nasa.gov/l3/order/). All correlations were significant at $p < 0.005$ (new Supplementary Fig. 3), making us confident that the ground-truthed relation is robust to the study region. In the revised manuscript we highlight that productivity was not measured but derived (Page 3, Lines 11-13). We also provide a more detailed explanation in the methods section (Page 9, Lines 7-16; new Supplementary Fig. 3) on how 'productivity' was derived and highlight the limitations of our approach (Page 9, Line 12). Overall, we believe that sea ice alone is insufficient to explain the observed changes in the benthos, and that productivity (which includes also insolation, Chl *a* and POC) is a useful measure for the environmental conditions affecting the benthos because it is wider than sea ice alone. We thus kept productivity as additional parameter to the sea ice.

- NASA Goddard Space Flight Center, Ocean Ecology Laboratory, Ocean Biology Processing Group. Sea-viewing Wide Field-of-view Sensor (SeaWiFS) Chlorophyll

Data. NASA OB.DAAC, Greenbelt, MD, USA. doi:10.5067/ORBVIEW-2/L3B/CHL/2018 (2018a).
- NASA Goddard Space Flight Center, Ocean Ecology Laboratory, Ocean Biology Processing Group. Sea-viewing Wide Field-of-view Sensor (SeaWiFS) Particulate Organic Carbon Data. NASA OB.DAAC, Greenbelt, MD, USA. doi:10.5067/ORBVIEW-2/L3B/POC/2018 (2018b).
- NASA Goddard Space Flight Center, Ocean Ecology Laboratory, Ocean Biology Processing Group. Moderate-resolution Imaging Spectroradiometer (MODIS) Aqua Chlorophyll Data. NASA OB.DAAC, Greenbelt, MD, USA. doi:10.5067/AQUA/MODIS/L3B/CHL/2018 (2018c).
- NASA Goddard Space Flight Center, Ocean Ecology Laboratory, Ocean Biology Processing Group. Moderate-resolution Imaging Spectroradiometer (MODIS) Aqua Particulate Organic Carbon Data. NASA OB.DAAC, Greenbelt, MD, USA. doi:10.5067/AQUA/MODIS/L3B/POC/2018 (2018d).

4) Iceberg scouring. As with productivity, impact frequency with the seabed was not measured or ground truthed. Their plots rely on number and area of icebergs, neither of which are strong predictors of seabed impact rate. Presence of icebergs doesn't indicate scouring, and area is not a great predictor of keel depth, is there any relevance of small icebergs in such depths (unlikely to be scouring). Look at seabed multibeam data - Scour density is often highest at shelf breaks - where there are few icebergs, but collisions are big because the seabed can be a bit shallower and icebergs gather more momentum with current and wind. Iceberg activity has been shown to decrease with 'whitening' because they can move around less [to collide with seabed]. Showing more icebergs does not imply more scouring. As with productivity it is implication rather than any direct measurement or ground trothing so I would delete this section, again not needed for the central story.

Reply: We agree with the reviewer that iceberg presence is not equivalent to scouring and that the frequency and size of icebergs are unlikely to be strong predictors of benthic disturbance. This is exactly what our analysis shows: impact frequency with the seabed was ground truthed in a semi-quantitative way by assessing the proportion of category 0 benthic assemblages in the megafaunal transects (fresh scours). The results indicate that more and larger icebergs had no direct effect on scouring (lower 3D configurations), but rather may have had indirect effects by consolidating sea-ice and reducing productivity, as the reviewer suggests. Because icebergs have frequently been shown to be important agents of benthic disturbance in shallow water (e.g. Dayton 1989, Gutt 2001, Barnes & Souster 2011), we find it important to consider the role of icebergs on deeper shelfer assemblages. For this reason, we would thus like to keep the icebergs in our manuscript.

In our revised text (Page 5, Lines 12-16) we summarize our findings on iceberg presence as follows: "As opposed to shallow coastal areas, where increased frequency and mobility of icebergs resulted in increased scouring of the ocean floor[16,43], the increase in icebergs in our study was not associated with a detectable increase in fresh scours (category 0; correlation $p < 0.05$, permutations = 9999). This suggests an indirect effect of icebergs[12,25] by reducing sea-ice motion, increasing sea-ice thickness and reducing primary productivity."

- Barnes, D.K.A. & Souster, T. Reduced survival of Antarctic benthos linked to climate-induced iceberg scouring. *Nat. Clim. Change.* **1**, 365–368. doi:10.1038/NCLIMATE1232 (2011).

- Dayton, P.K. Interdecadal variation in an Antarctic sponge and its predators from oceanographic climate shifts. *Science* **245**, 1484–1486. doi:10.1126/science.245.4925.1484 (1989).
- Gutt, J. On the direct impact of ice on marine benthic communities, a review. *Polar Biol.* **24**, 553–564. doi:10.1007/s003000100262 (2001).

Minor points
P1 The title '..on a whitening Antarctic continental shelf.', abstract 'In much of the East Antarctic, however, sea-ice has increased over the last decades' and start of the discussion are misleading. As the authors say later that 'increase' in sea ice has since been lost, and the net loss around Antarctica is now the same as 30 years of loss around the Arctic.

Reply: The sea-ice increase refers to the study period (1988-2014), and does not lead up to the present (1988-2020). We clarified this in the revised abstract and discussion. Page 1, Lines 9-14 of the revised abstract now read as follows:

"Ice retreat in West Antarctica and Antarctic Peninsula has led to important changes in seafloor communities and gains in benthic blue carbon. In much of the East Antarctic, however, sea ice has increased from 1988 to 2014, but its effects on the benthos remain largely unexplored. Here, we provide a 26-year record of macro- and megafauna from the northeastern Weddell Sea shelf where benthic biomass decreased by two-thirds and composition shifted from suspension feeders to deposit feeders."

We re-wrote Page 5, Lines 19-21 in the Discussion as follows:

"Our study, spanning more than a quarter of a century, is the first to the best of our knowledge, showing quantitative benthic community changes over the full depth of the continental shelf. It is also the first to examine benthic changes in a region of the Antarctic that has been whitening until very recently…"

P2 Remove 'The lack of crushing predators has led to a rich epifauna dominated by sessile suspension feeders'? it does not add to the argument and is contentious. There is certainly 'a rich epifauna dominated by sessile suspension feeders' around Antarctica, whether it is due to rarity of crushing predators is opinion. There are other places dominated by sessile, primary consumers (inc suspension feeders) that have duraophagus predators.
Change '..carbon captured by benthos' to '..carbon stored by benthos'.

Reply: As suggested, we removed the sentence "The lack of crushing predators has led to a rich epifauna dominated by sessile suspension feeders". We have also modified "...carbon captured by benthos" to "… carbon stored by benthos".

P5 and throughout change 'community' to 'assemblage'. Community refers to the foodweb with all of its components, the authors just measure part of that community – the macro and megabenthos.

Reply: We use the terminology proposed by Odum & Barrett (1971) which is widely used in ecology (>13,000 citations). They use organism, population, community as subsets to the ecosystem (and landscape, biome, and ecosphere suprasets). The term 'assemblage' applies to taxonomically related populations (Stroud et al. 2015) and is thus inappropriate in our context. In addressing the taxonomically unrelated large seafloor fauna, we thus adhere to the "macro- and megabenthos community" terminology of our original manuscript.

- Odum, E. P., & Barrett, G. W. (1971). *Fundamentals of ecology* (Vol. 3, p. 5). Philadelphia: Saunders.
- Stroud, J. T., Bush, M. R., Ladd, M. C., Nowicki, R. J., Shantz, A. A., & Sweatman, J. (2015). Is a community still a community? Reviewing definitions of key terms in community ecology. *Ecol. Evol.*, **5(21)**, 4757-4765.

- Change 'Our results provide important ground-truthing of the proposed links between productivity, iceberg presence and benthic abundance and biomass' to 'Our results provide important ground-truthing of the proposed links between sea ice and iceberg presence and benthic biomass' (ie delete productivity and abundance [the latter since no relationship is demonstrated], but include sea ice.

Reply: We changed the sentence to include all findings (i.e. both, the significant and the non-significant results) as follows (Page 5, Lines 19-23):

"Our study, spanning more than a quarter of a century, is the first to the best of our knowledge, showing quantitative benthic community changes over the full depth of the continental shelf. It is also the first to examine benthic changes in a region of the Antarctic that has been whitening until very recently, thus providing an important test of the proposed links between sea ice and iceberg cover and benthic abundance and biomass[44]. ".

- Delete 'and refocus the attention to the larger part of the Antarctic continental shelf that has been whitening: only the Bellingshausen/Amundsen sector of the Antarctic has seen a negative trend in sea-ice extent over 40 years of satellite observations, whereas the remainder of the Antarctic has seen increasing trends in sea-ice extent so that, with the exception of the Bellingshausen/ Amundsen sector, an overall decrease in blue carbon can be expected.' Firstly the Scotia sea consistently lost sea ice as well, but recent papers eg Turner & Comiso 2017 show virtually all sectors have now lost net sea ice over that period.

Reply: We removed the geographical detail as it is not relevant to the study. We re-wrote the sentence in general terms as follows (Page 5, Lines 23-25):

"Because most of the Antarctic continental shelf has seen a positive trend in sea-ice extent over 40 years of satellite observations until 2014[28,45], an overall decrease in blue carbon can be expected."

- I'm not convinced that 'Extrapolating our findings from the KNA area (Fig. 2) to whitening Antarctic shelves we calculate a 31.3 x 106 t km-2 blue carbon loss over the study period' is valid (even assuming that the trend stays true with recalculated replication). It is certainly a loss of 'blue carbon storage' but some of that may be buried in berms by icebergs. The key value of blue carbon as an ecosystem service is removal from the carbon cycle, ie burial to sequestration, which is much more 'valuable' than blue carbon storage in living benthos. Examining the organic carbon subsurface profile in multicores would be able to confirm whether it is actually a net loss.. or whether much of that blue carbon storage has been converted to sequestered carbon. The authors could make the sentence true by inserting the words 'zoobenthic' before and 'storage' after blue carbon but I think that would be disingenuous without the corroborating geochemistry from core profiles to support it; otherwise delete it, the ms is still okay without it.

Reply: We agree with the reviewer. The revised manuscript now reads (Page 5, Line 25-Page 6, Line 3):

“If corroborated by geochemical evidence, the extrapolation of our findings to the total Antarctic continental shelf would imply an overall decrease in Antarctic zoobenthic blue carbon storage of 31.3×10^6 t km^{-2} during that period, contrasting the situation described for the West Antarctic and Antarctic Peninsula region where blue carbon has been increasing in response to climate change[17,18,20,21]”.

- As a general comment, not all blue carbon is equal - if the point of evaluating it is to assess an ‘societal ecosystem service’. The authors could make their data more valuable to assessment of blue carbon by splitting their groups of fauna up more from merely feeding guilds. On death, the carbon in soft bodied forms such as an errant polychaete worm are nearly all entirely recycled in the microbial loop. In contrast carbon within heavily skeletalised forms are much more likely to sequester that carbon (hence their more obvious fossil record).

Reply: This is an interesting new aspect worthy of study. It exceeds, however, the scope of this paper.

P7 The box cores sample a precise area of seabed, not so most cameras. Please include more detail on the nature and error associated with the imagery used. What angles were the camera and video to the seabed? How large was the field of view and the area of analysis within each imagery sample? Importantly, how was area within image calculated? Please provide an extra suppl table showing levels of error of area calculation within the image and video samples. The reader should be informed about data reliability [extracted from imagery].

Reply: As mentioned earlier, the megafauna in the underwater images was not quantified, but broadly categorized. The suggested parameters (camera angle, areas, etc.) are not necessary to assess the broad categorizations undertaken in this study.

P9 The iceberg methodology and data is weak and unsubstantiated so I would remove.

Reply: We improved the iceberg methodology and data by using Altiberg version 2.1 (Tournadre et al. 2017), an updated version of the Tournadre et al. (2016) paper in the original submission. It contains the intercalibrated and merged data set from ten different altimeters, including also the Geosat 1986-1989 data, which now provides a homogeneous climatology for the study region with strong data on the probability of presence, mean surface and volume of iceberg over the study period. This allowed us to avoid transformations which may have weakened the earlier data set. Our correlation analysis on the revised data set is in line with our original findings, suggesting an indirect effect of icebergs on the benthos. We consider it important to evaluate direct versus indirect effects and keep the icebergs as potentially important factor, even though the final results show that no direct effect on scouring are evident. Thus, we decided not to remove the icebergs in the revised manuscript.

- Tournadre, J., Bouhier, N., Girard-Ardhuin F. & Rémy, F. Antarctic icebergs distributions 1992-2014. *J. Geophys. Res.-Oceans.* **121**, 327–349. doi:10.1002/2015JC011178 (2016).
- Tournadre, J., Accensi, M. & Girard-Ardhuin F. *The ALTIBERG iceberg data base version-2.1.* (Ifremer, 2019)

REVIEWER'S COMMENTS:

Reviewer #1 (Remarks to the Author):

You easily satisfied my comments in your letter and in the manuscript. The other reviewer, however, did an extremely careful job and caught things I either missed or did not concern me. You are lucky to get such a careful reviewer and in places you struggled to answer all the concerns, largely because of space limitations. You various solutions, compromises and explanations satisfied me, but the other reviewer will need to address their perspective.

We both wanted a lot more information than you have space to offer and I sincerely hope you can get out a much more complete paper soon, especially given that the ice situation is now changing for the worse, so this study will be very important.

Paul Dayton

Reviewer #2 (Remarks to the Author):

The authors have worked well to revise a manuscript that I think now merits acceptance with minor corrections. I think they still need to remove icescouring throughout the ms as they did not measure it, merely inferred it from a lack of fauna. I have a few suggested changes to complete the revised version

1) Title change 'declines' to 'declined'
2) Abstract line 10 change '..sea ice has increased..' to '..sea ice increased..'
3) Abstract line 14 change 'Concomitant increases in sea-ice and icebergs suggest..' to 'Concomitant increases in sea-ice suggests..' There was no significant increase in icebergs.
4) line 57 change 'we analysed 98 imagery transects (see Methods), to assess iceberg scours and the megafauna..' to 'we analysed 98 imagery transects (see Methods), to assess megafauna..' All icescour assessment appears to have been done entirely by inference that there is no fauna there - this is not the same as measuring it.
5)Line 133 change 'Most Antarctic benthic species are stenothermal..' to 'Most of the few Antarctic benthic species to be investigated are stenothermal..'

Figure 1 remove grey 'non significant regression lines' - a non significant regression = no relationship.
Figure 4 change 'Fresh scour' to 'No fauna' unless you have a separate line of evidence that it was actually a scour in each case (eg multibeam of scour morphology). Likewise removing 'scouring anomalies' from line 171 and 213.
Figure 5 remove the green '+' boxes, change to grey. Significance is <0.05, above this is simply non significant (ie no change).

Well done, good effort on the science and revision.

David K A Barnes

POINT-BY-POINT REPLY TO REVIEWER'S COMMENTS:

Reviewer #1 (Remarks to the Author):

You easily satisfied my comments in your letter and in the manuscript. The other reviewer, however, did an extremely careful job and caught things I either missed or did not concern me. You are lucky to get such a careful reviewer and in places you struggled to answer all the concerns, largely because of space limitations. You various solutions, compromises and explanations satisfied me, but the other reviewer will need to address their perspective.

We both wanted a lot more information than you have space to offer and I sincerely hope you can get out a much more complete paper soon, especially given that the ice situation is now changing for the worse, so this study will be very important.

Paul Dayton

Reply: We are grateful to both, Paul Dayton's and David Barnes' authoritative comments and insightful suggestions which have greatly helped us to improve our paper.

We will follow up on the suggestion to provide a more detailed account elsewhere. We will also use the opportunity of a research cruise to the Weddell Sea scheduled for early 2021 to re-visit the KNA area and see how the benthos has changed since the sea-ice maximum in 2014, when we last sampled the area.

Reviewer #2 (Remarks to the Author):

The authors have worked well to revise a manuscript that I think now merits acceptance with minor corrections. I think they still need to remove ice scouring throughout the ms as they did not measure it, merely inferred it from a lack of fauna. I have a few suggested changes to complete the revised version

Reply: We thank David Barnes for his very careful and constructive revision and the many important suggestions and comments which greatly helped to improve the manuscript. It is true that we did not measure iceberg scouring. It is also true that lack of epifauna alone is insufficient to infer iceberg scouring. However, the combination of both, lack of fauna *and* abrupt change in benthic cover and size on either side of the putative disturbance are now widely accepted criteria for identifying iceberg scours in underwater imagery, as established e.g. by Gutt (2001) and Gutt & Starmans (2001). We added the important aspect of spatial heterogeneity in the revised ms, which now reads (lines 172-176 of the clean manuscript):

Category 0 represented a putative scour mark in its earliest stage of recolonization, characterized by a lack of megafauna and an abrupt change in benthic cover and size

along the transect (equivalent to stage R0 in Gutt & Starmans[34]). Categories 1 to 5 represent later stages of colonization by Antarctic shelf megafaunal assemblages sensu Gili et al.[57], of increasing three-dimensionality (see Fig. 1, in Gili et al.[57]).

1) Title change 'declines' to 'declined'

Reply: Done. The title now reads “Benthic fauna declined on a whitening Antarctic continental shelf” (Line 1 of the clean manuscript).

2) Abstract line 10 change '..sea ice has increased..' to '..sea ice increased..'

Reply: Done (Lines 10 of the clean manuscript).

3) Abstract line 14 change 'Concomitant increases in sea-ice and icebergs suggest..' to 'Concomitant increases in sea-ice suggests..' There was no significant increase in icebergs.

Reply: Done (Lines 14 of the clean manuscript).

4) Line 57 change 'we analyzed 98 imagery transects (see Methods), to assess iceberg scours and the megafauna..' to 'we analyzed 98 imagery transects (see Methods), to assess megafauna..' All ice scour assessment appears to have been done entirely by inference that there is no fauna there - this is not the same as measuring it.

Reply: As mentioned earlier, the ice scour assessments were done by inference that there is no fauna there and the spatial changes are abrupt. We acknowledge the lack of direct evidence and modified the sentence to soften the putative link between megafauna and scouring, so that it now reads “… Additionally, we analysed 98 imagery transects (see Methods), to assess the megafauna which is not quantitatively represented in the box cores and whose complexity varies in response to iceberg disturbance …” (Lines 56-58 of the clean manuscript).

5) Line 133 change 'Most Antarctic benthic species are stenothermal..' to 'Most of the few Antarctic benthic species to be investigated are stenothermal..'

Reply: Done (Lines 135-136 of the clean manuscript).

Figure 1 remove grey 'non significant regression lines' - a non significant regression = no relationship.

Reply: We removed all non-significant regression lines, and modified the figure caption accordingly.

Figure 4 change 'Fresh scour' to 'No fauna' unless you have a separate line of evidence that it was actually a scour in each case (eg multibeam of scour morphology). Likewise removing 'scouring anomalies' from line 171 and 213.

Reply: As mentioned above, scours were identified as areas devoid of megafauna bordered by abrupt changes in megafauna cover and size. We thus felt it appropriate to keep the “Fresh scour” terminology in the revised ms. We removed “scouring anomalies” in the revised ms.

Figure 5 remove the green '+' boxes, change to grey. Significance is <0.05, above this is simply non significant (ie no change).

Reply: Done. We modified our Fig. 5 and changed all “+” boxes to grey. Trend directions were colour-coded (green: positive, red: negative).

Well done, good effort on the science and revision.

David K A Barnes

Reply: We thank David Barnes again for his time and painstaking review which have helped us a lot in improving our manuscript.
